# *Xrcc6* coordinates cardiomyocyte repair and immune regulation in myocardial ischemia-reperfusion injury: Fisetin as a therapeutic modulator

**DOI:** 10.3389/fimmu.2025.1653738

**Published:** 2025-09-17

**Authors:** Yijie He, Jin Li, Linlong Guo, Mu Chen, Haiqing Pan, Zhanqing Li, Hua Tian, Huan Yu, Yuhong Zhou, Hongwen Xiao

**Affiliations:** ^1^ Department of Basic Medicine, Key Laboratory Of Functional and Clinical Translational Medicine, Xiamen Medical College, Xiamen, Fujian, China; ^2^ Department of Basic Medicine, Institute of Respiratory Diseases Xiamen Medical College of Respiratory Diseases, Xiamen Medical College, Xiamen, Fujian, China; ^3^ Harbin Medical University, Harbin, Heilongjiang, China

**Keywords:** ischemia-reperfusion, XRCC6, Fisetin, molecular dynamics, DNA damage

## Abstract

**Introduction:**

Myocardial ischemia-reperfusion (I/R) injury remains a major challenge in the treatment of ischemic heart disease. The DNA damage repair gene Xrcc6 has been implicated in cardiovascular pathology, but its role in myocardial I/R injury and its regulation by natural compounds remains unclear. We aimed to elucidate the role of Xrcc6 in I/R injury and to investigate the cardioprotective effects of the flavonoid Fisetin through Xrcc6-targeted modulation.

**Methods:**

We integrated bulk and single-cell RNA sequencing to analyze cardiomyocyte subtypes and gene expression profiles, and constructed co-expression modules using high-dimensional weighted gene co-expression network analysis (hdWGCNA). Trajectory inference and intercellular communication analyses were performed to assess cell fate dynamics and immune regulation. Molecular docking and dynamics simulations were used to evaluate Fisetin–Xrcc6 interactions. In vivo murine models of I/R injury were employed to confirm transcriptomic findings and to assess Fisetin’s cardioprotective mechanisms.

**Results:**

Transcriptomic analysis revealed significant downregulation of Xrcc6 post-I/R, with single-cell data highlighting vCMs3 as a reparative cardiomyocyte subtype whose abundance correlated with Xrcc6 expression. Pseudotime analysis positioned vCMs3 at early differentiation stages with dynamic Xrcc6 expression along the trajectory. CIBERSORT and CellChat linked Xrcc6 to macrophage polarization and immune regulation. Docking simulations demonstrated stable Fisetin–Xrcc6 binding (binding free energy: −7.55 kcal/mol). In vivo, Fisetin upregulated Xrcc6, reduced DNA damage (γH2A.X suppression), modulated inflammatory responses, and improved cardiac function after I/R injury.

**Discussion:**

Our study identifies Xrcc6 as a dual regulator of cardiomyocyte fate determination and immune modulation during myocardial I/R injury. Fisetin confers cardioprotection by targeting Xrcc6, offering mechanistic insights into DNA repair–immune crosstalk and providing a potential therapeutic strategy for ischemic heart disease.

## 1 Introduction

Cardiovascular diseases (CVDs) remain one of the leading causes of morbidity and mortality worldwide, with ischemic heart disease (IHD) contributing significantly to this burden (1–3). Despite advances in interventional therapies such as percutaneous coronary intervention (PCI) (4) and thrombolytic therapy (5) that have markedly improved the short-term prognosis of patients with myocardial infarction, myocardial ischemia-reperfusion (I/R) injury (6)—caused by the restoration of blood flow—has emerged as a critical barrier to long-term cardiac functional recovery and quality of life. Myocardial I/R injury triggers a cascade of pathological events, including oxidative stress (7), inflammatory responses (8), mitochondrial dysfunction (9), calcium overload, and various forms of programmed cell death, all of which compromise the structural and functional integrity of the myocardium (10). Elucidating the molecular mechanisms underlying myocardial I/R injury and identifying effective therapeutic targets are therefore key priorities in cardiovascular basic and translational research.

Among the multifactorial mechanisms implicated in I/R injury, oxidative stress-induced DNA damage (11)—particularly DNA double-strand breaks (DSBs)—plays a pivotal role in cardiomyocyte death and dysfunction (12, 13). DSBs represent one of the most lethal forms of DNA damage, directly threatening genomic stability and cell viability (14). Studies have shown that during ischemia and subsequent reperfusion, the excessive oxidative environment leads to significant accumulation of DNA damage in cardiomyocytes, resulting in p53 activation (15), cell cycle arrest, apoptosis, and necrosis (13). The maintenance of genomic integrity depends on the DNA damage response system, with non-homologous end joining (NHEJ) (16) and homologous recombination (HR) (17) serving as the principal pathways for DSB repair. *Xrcc6* (X-ray repair cross-complementing protein 6, also known as Ku70) is a key component of the NHEJ pathway. It forms a heterodimer with Ku80 to recognize broken DNA ends and recruit downstream proteins such as DNA-PKcs to facilitate repair (18, 19). While *Xrcc6* has been shown to be essential for DNA repair and cell survival in various cell types, its expression dynamics, cell-type specificity, and regulatory mechanisms in the context of myocardial I/R injury remain poorly understood (20).

The recent emergence of single-cell RNA sequencing (snRNA-seq) has provided powerful tools to dissect cellular heterogeneity within cardiac tissue under physiological and pathological conditions. Unlike traditional bulk RNA sequencing, snRNA-seq enables the identification of distinct cell subpopulations, as well as the tracking of their transcriptional states and fate trajectories at single-cell resolution (21). In the setting of myocardial I/R injury, the heterogeneity of cardiomyocytes, their differentiation potential, and the functional reprogramming involved in tissue repair have yet to be systematically characterized. Moreover, I/R injury not only affects cardiomyocytes but also reshapes the cardiac immune microenvironment, notably altering macrophage polarization states (22). Immune cell activation and inflammatory responses significantly influence cardiomyocyte survival and tissue regeneration (23). Thus, understanding how immune cell function is modulated during I/R injury—particularly through DNA repair factors—has become an important area of investigation.

Natural compounds derived from traditional Chinese medicine have attracted increasing attention in cardiovascular research due to their multi-target effects, low toxicity, and oral availability (24). Fisetin, a natural flavonoid (25) found in plants such as Rhus, strawberries, apples, and onions, exhibits a broad range of biological activities, including antioxidant, anti-inflammatory, anti-aging, and anti-tumor effects (26). Previous studies have demonstrated that Fisetin protects against tissue injury in various models by scavenging free radicals, inhibiting NF-κB signaling, and modulating apoptotic pathways (27). However, its cardioprotective potential in myocardial I/R injury and its molecular targets remain largely unexplored, particularly with respect to whether it can exert protective effects through the regulation of DNA repair pathways (28).

Given this background, the objectives of the present study are threefold: (1) to investigate the role of *Xrcc6* in myocardial I/R injury and its critical functions in cardiomyocyte repair; (2) to examine changes in cardiomyocyte subtypes during I/R injury and the regulatory role of *Xrcc6* using single-cell transcriptomics; and (3) to evaluate the cardioprotective effects of Fisetin in I/R models and elucidate its underlying mechanisms, particularly whether it acts through modulation of *Xrcc6*. Collectively, this research aims to provide novel mechanistic insights and identify targeted strategies for the treatment of myocardial I/R injury, while advancing the therapeutic potential of natural compounds in cardiovascular disease.

## 2 Methods

### 2.1 Establishment of an *in vitro* ischemia-reperfusion injury model in primary cardiomyocytes

To simulate myocardial ischemia-reperfusion (I/R) injury *in vitro*, an oxidative stress model was established using hydrogen peroxide (H_22_)-induced injury in primary neonatal mouse cardiomyocytes. Neonatal C57BL/6 mice (1–3 days old) were euthanized under sterile conditions, and the hearts were rapidly excised and rinsed in ice-cold phosphate-buffered saline (PBS) to remove residual blood. The cardiac tissue was then enzymatically dissociated using a combination of trypsin and collagenase to obtain a single-cell suspension.

The isolated cardiomyocytes were filtered through a cell strainer, centrifuged, and seeded onto culture dishes pre-coated with 0.1% poly-L-lysine. Cells were maintained in DMEM/F12 medium supplemented with 10% fetal bovine serum (FBS) and incubated at 37°C in a humidified atmosphere containing 5% CO_2_. Once the cells adhered and reached the logarithmic growth phase, they were subjected to oxidative stress treatment.

To model oxidative injury associated with I/R, cardiomyocytes were incubated in serum-free medium containing 100 μM H_22_ for 24 hours, based on preliminary optimization experiments. This exposure mimicked transient oxidative stress during ischemia. Subsequently, the medium was replaced with standard culture medium, and cells were maintained for an additional 24 hours to simulate the reperfusion phase.

### 2.2 Bulk RNA sequencing of primary cardiomyocytes undergoing ischemia-reperfusion injury

Total RNA was extracted from cardiomyocytes using TRIzol reagent (Invitrogen, USA) according to the manufacturer’s instructions. RNA purity and concentration were assessed using a NanoDrop 2000 spectrophotometer (Thermo Scientific, USA), while RNA integrity was evaluated using an Agilent 2100 Bioanalyzer (Agilent Technologies, Santa Clara, CA, USA). Transcriptome libraries were constructed using the VAHTS Universal V5 RNA-seq Library Prep Kit (Vazyme, China), following the manufacturer’s protocol. Bulk RNA sequencing and subsequent data analysis were conducted by OE Biotech Co., Ltd. (Shanghai, China).

#### 2.2.1 Identification and functional characterization of DNA damage repair genes via multi-database integration

To systematically identify genes involved in DNA damage repair (DDR), we integrated multiple curated databases. Specifically, we intersected gene sets from the Human DNA Repair Genes database (29), the DNA repair-related gene sets in the Molecular Signatures Database (MsigDB), and DDR-associated genes from the GeneCards database. This approach yielded a high-confidence core set of 220 DDR genes (**Supplementary Table 1**).

For sample clustering analysis, principal component analysis (PCA) was performed. Gene expression data were first normalized using z-score transformation. Dimensionality reduction was then conducted using the *prcomp* function from the *stats* package in R (version 3.6.0), allowing for visualization of sample distribution along principal components.

Differential expression analysis was carried out using the *DESeq2* package (version 1.32.0) (30). Genes with zero expression in more than 80% of samples were excluded during preprocessing. Count matrices were generated using the DESeqDataSetFromMatrix function, normalized using the DESeq function, and differential expression statistics were obtained via the results function.

To explore the biological functions of differentially expressed genes (DEGs), Gene Ontology (GO) enrichment analysis was performed using the *clusterProfiler* package (version 3.14.3) (31), with gene annotations from *org.Hs.eg.db* (version 3.1.0) as background. Analysis parameters were set to a gene set size between 5 and 5000, with significance thresholds of *p* < 0.05 and false discovery rate (FDR) < 0.25.

#### 2.2.2 Immune cell infiltration analysis

CIBERSORT (32), a widely adopted tool for immune cell deconvolution, employs a linear support vector regression algorithm based on a reference gene expression matrix of 22 immune cell subtypes (LM22). In this study, the *CIBERSORT* algorithm was applied to RNA-seq data to quantitatively estimate the relative abundance of 22 immune cell types within each sample.

To further investigate the relationship between gene expression and immune infiltration, Spearman correlation analysis was conducted between the expression of key genes and the estimated proportions of immune cell subtypes. Results were visualized using hierarchical clustering heatmaps and scatter plots, enabling a comprehensive characterization of the immune landscape and its association with gene expression patterns in the I/R injury microenvironment.

### 2.3 Single-cell RNA sequencing analysis of cardiac tissue from a mouse ischemia-reperfusion injury model

#### 2.3.1 Data quality control and preprocessing

Stringent quality control (QC) criteria were applied to preprocess single-cell RNA sequencing (snRNA-seq) data. The following thresholds were used to filter out low-quality cells while retaining biologically informative populations (1): mitochondrial gene expression percentage < 30%; (2) minimum number of unique molecular identifiers per cell ≥ 500; (3) minimum number of detected genes per cell ≥ 250.To reduce technical artifacts, a multi-step QC pipeline was employed: (1)Standardization of all samples using the *omicverse* analytical framework (33, 34). (2)Detection and removal of potential doublets using the *sccomposite* algorithm (35) to eliminate cell multiplets that could confound downstream analyses. (3)Batch effect correction across samples using the *Harmony* algorithm (36), ensuring the comparability of datasets from different biological replicates or experimental conditions. The outcomes of Harmony integration, visualized through UMAP projections before and after correction, confirmed effective removal of batch effects while preserving biologically meaningful clustering (**Supplementary Figure S2**).

Detailed metadata for all samples, including GEO accession IDs, treatment conditions, and sequencing parameters, are provided in **Supplementary Table 2**.

#### 2.3.2 Dimensionality reduction, clustering, and cell type annotation

Following batch correction, snRNA-seq data were subjected to dimensionality reduction, unsupervised clustering, and cell type identification. The first 50 Harmony-corrected principal components were used to construct a k-nearest neighbors (k-NN) graph (k = 15), representing cellular similarity. Non-linear dimensionality reduction was then performed using the UMAP algorithm for visualization of the cellular landscape.

Unsupervised clustering was conducted using the *Leiden* algorithm (37), with the clustering resolution optimized to identify distinct and biologically meaningful cell subpopulations. Cell type annotation was carried out through a hierarchical strategy integrating multiple lines of evidence: Expression patterns of known marker genes, QC metrics, and Curated reference data from the CellMarker 2.0 database.

Each cell cluster was manually annotated based on this integrated approach to ensure high-confidence classification.

#### 2.3.3 Identification of cardiomyocyte subtypes

From the clustered dataset, cardiomyocytes were extracted for focused analysis. These cells were subjected to a separate round of Leiden clustering to identify putative cardiomyocyte subtypes.

To validate the biological relevance of each subtype, the following analyses were conducted: (1) Identification of subtype-specific differentially expressed genes (DEGs) using thresholds of *log FC* > 0.25 and *p* < 0.05. (2) GO biological process enrichment analysis of DEGs using the *clusterProfiler* package (*p.adjust* < 0.05). (3) Functional annotation based on known cardiomyocyte development and functional pathways, such as cardiac contraction and energy metabolism.

Finally, UMAP visualization was used to map the spatial distribution of distinct functional subtypes. The relative abundance of each subtype was quantified under different experimental conditions to reveal dynamic changes associated with ischemia-reperfusion injury.

#### 2.3.4 Abundance analysis of cardiomyocyte subpopulations

To systematically assess the dynamic changes in cardiomyocyte subpopulation abundance, we applied the *miloR* algorithm (38). The Seurat object containing single-cell RNA-seq data was first converted into a SingleCellExperiment object while retaining the original dimensionality reduction results. This object was then used to initialize the Milo analysis framework.

Using the top 50 principal components, we constructed a k-nearest neighbor graph and defined cell neighborhoods using a refined strategy. For each sample, the distribution of cells across neighborhoods was computed. A generalized linear model (GLM) was applied to test for differences in neighborhood abundance between conditions, and a distance matrix was computed to evaluate the spatial relationships among neighborhoods. The statistical significance threshold was set at *P* < 0.05.

Significantly altered neighborhoods (|logFC| > 1) were visualized by projecting them onto the UMAP embedding. Swarm plots were used to depict logFC changes for each cardiomyocyte subpopulation, where blue indicates enrichment in the normal control (NC) group and red indicates enrichment in the ischemia-reperfusion (I/R) group. All plots were generated and optimized using the ggplot2 package. This approach provided a high-resolution view of compositional shifts in cardiomyocyte populations under pathological conditions, offering new insights into cellular heterogeneity during cardiac injury.

#### 2.3.5 hdWGCNA analysis of cardiomyocyte subpopulations and key gene identification

To explore the regulatory architecture of gene expression across cardiomyocyte subtypes, we employed high-dimensional weighted gene co-expression network analysis (39). This method allowed us to construct and analyze co-expression networks to identify key gene modules within specific subpopulations.

The analysis pipeline was as follows: First, snRNA-seq data were preprocessed, and meta cells were constructed based on UMAP embeddings to reduce data sparsity. The expression matrix was normalized, and genes expressed in at least 5% of cells were retained using the “fraction” filtering method. A soft-thresholding power was determined to ensure scale-free topology, followed by the construction of a signed topological overlap matrix (TOM) and hierarchical clustering to define gene modules.

To assess the biological relevance of each module, we calculated module eigengenes (hMEs) and gene connectivity scores (kME). The top 25 hub genes from each module were selected for downstream analysis. Module scores were computed using the *UCell* algorithm, and their distribution across cardiomyocyte subtypes was visualized using UMAP projections.

Additionally, marker genes for each cardiomyocyte subtype were identified using Seurat’s FindAllMarkers function. Overlap between module genes and cell-type marker genes was quantified using the *GeneOverlap* package, providing insight into subtype-specific regulatory modules. All figures were created using the *ggplot2* and *patchwork* packages.

To identify functional key genes, we intersected the list of differentially expressed DNA damage response related genes (derived from bulk RNA-seq) with hub genes identified in the hdWGCNA network. This Venn-based integration strategy pinpointed candidate genes that were both significantly dysregulated and centrally positioned within co-expression networks. These genes were considered key regulators of cardiomyocyte function in the context of I/R injury. By combining differential expression analysis with network centrality, this strategy enhances the robustness and biological relevance of subsequent functional validation efforts.

#### 2.3.6 Pseudotime analysis of cardiomyocyte subpopulations

To investigate the dynamic trajectories of cardiomyocyte development, we employed a multi-step integrative pseudotime analysis strategy. Initially, *CytoTRACE2* (40) was used to infer cellular differentiation potential. Key parameters included a batch size of 10,000, a smoothing window of 1,000, a maximum of 50 principal components, and a fixed random seed (random_state = 14). Parallel computation was enabled to ensure robustness and computational efficiency.

Using the UMAP embedding and the batch-corrected principal component matrix (X_pca_harmony), the top 50 principal components were used as input features. Trajectory root points were determined based on CytoTRACE2-inferred differentiation scores. To construct developmental lineages, we applied the *Slingshot* algorithm (41) with three iterative refinements (n_epochs = 10), enhancing the accuracy of inferred paths. We further employed the *PAGA* (Partition-based Graph Abstraction) algorithm to explore topological relationships among cell states.

For high-resolution trajectory sampling, we integrated the *Palantir* algorithm (42), setting the number of waypoints to 500. This approach allowed for the calculation of branch probabilities and pseudotime entropy, offering insights into the dynamics of cell fate decisions.

To resolve complex trajectory structures such as branching and cyclic transitions, we also utilized the *VIA* algorithm (43). VIA combines graph neural networks with MCMC-based optimization and features a novel “delayed jump” random walk mechanism, demonstrating high robustness in modeling non-linear and multifurcating trajectories.

All gene expression matrices used in trajectory analysis were first imputed using the *MAGIC* algorithm to reduce sparsity. Pseudotime trajectories generated by different methods were integrated to examine temporal expression dynamics of key genes. Results were visualized in UMAP space, mapped alongside cell-type annotations, enabling multidimensional validation and interpretation.

#### 2.3.7 Cell–cell communication analysis

To investigate differences in intercellular communication networks under distinct experimental conditions, we performed systematic cell–cell interaction analysis using the *CellChat* (44) framework in R.

Independent CellChat objects were constructed for both the normal control (NC) and ischemia-reperfusion (I/R) groups. The computeCommunProb function was used to quantify communication probabilities between cell subpopulations, while the computeCommunProbPathway function was employed to identify specific signaling pathway interactions.

The ligand–receptor interaction database was sourced from *CellChatDB.mouse*, which contains a comprehensive set of curated mouse ligand–receptor pairs. To ensure the reliability of inferred interactions, a minimum cell count threshold of *n* = 10 was applied to filter out low-confidence communication events.

This analysis enabled a comparative view of intercellular signaling landscapes between physiological and pathological states, providing mechanistic insights into how cardiomyocyte and non-cardiomyocyte populations coordinate during I/R injury.

### 2.4 Molecular docking and molecular dynamics simulation of Fisetin with *Xrcc6*

#### 2.4.1 Molecular docking

To investigate the potential binding mode between Fisetin and the *Xrcc6* protein (45), molecular docking was performed using computational approaches. The 3D structure of Fisetin was retrieved from the PubChem database, and docking simulations were carried out with *AutoDock Vina* (v1.2.0) (46). First, the active binding pockets of Xrcc6 were predicted using the *CB-DOCK2* algorithm (47), and the corresponding spatial coordinates were selected as the docking targets.

Subsequently, the docking was refined using Vina’s scoring function to predict the optimal binding conformation of Fisetin at the active site. The binding free energy was calculated to evaluate the interaction strength, providing a reliable structural basis for the following molecular dynamics (MD) simulations.

#### 2.4.2 Molecular dynamics simulation

To further assess the stability of the Fisetin–Xrcc6 interaction, molecular dynamics simulations were conducted using *GROMACS 2022* (48). The AMBER14SB force field was applied to the protein, and the GAFF (General AMBER Force Field) was used for the Fisetin ligand, as the AMBER framework offers accurate protein backbone energetics, reliable ligand parametrization, and has been widely validated in protein–ligand binding free-energy simulations (49–52). The system was solvated in a TIP3P water box under periodic boundary conditions. Following energy minimization, equilibration was conducted through 100 ps of NVT and 100 ps of NPT simulations to ensure structural stability. LINCS constraints were applied to all hydrogen bonds with a 2 fs integration time step. Long-range electrostatics were treated using the Particle Mesh Ewald (PME) method with a cutoff of 1.2 nm, and van der Waals interactions were truncated at 10 Å with the neighbor list updated every 10 steps. The system temperature was maintained at 298 K using the V-rescale thermostat, and the pressure was kept at 1 bar using the Berendsen barostat. A 100 ns production run was then carried out, with trajectory snapshots saved every 10 ps for further analysis. Visualization and structural evaluations were performed using VMD and PyMOL, focusing on root mean square deviation (RMSD) and the dynamic profile of hydrogen-bond formation throughout the simulation. Finally, binding free energy was estimated with the MM/PBSA method using the g_mmpbsa tool, providing additional evidence for the stability and potential biological relevance of the Fisetin–Xrcc6 interaction.

### 2.5 Protective effects and mechanistic validation of Fisetin on myocardial ischemia-reperfusion injury

#### 2.5.1 Animals and treatment

**Figure 1:**
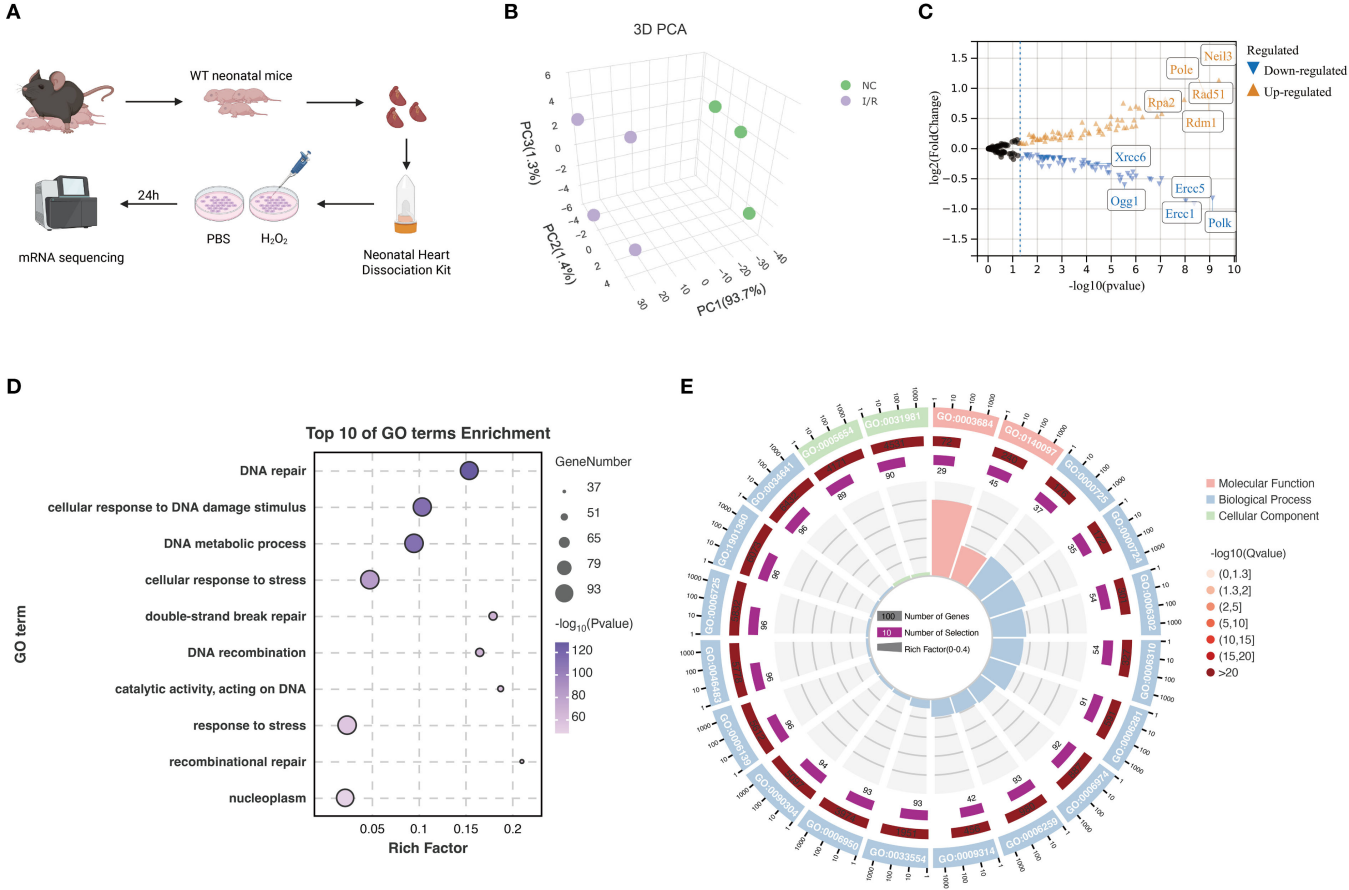
Transcriptomic profiling of mRNA expression changes in primary mouse cardiomyocytes. **(A)** Schematic diagram of the experimental workflow. Cardiac tissues were isolated from neonatal wild-type mice, and cardiomyocytes were dissociated using a commercial cardiac cell dissociation kit. Cells were treated with PBS or H_22_ for 24 hours. Total RNA was extracted and subjected to mRNA sequencing. **(B)** 3D principal component analysis (PCA) plot showing clear separation between the NC and I/R groups, indicating significant differences in mRNA expression profiles. **(C)** Volcano plot of differentially expressed genes. Upregulated genes are shown in orange and downregulated genes in blue. Significantly altered genes such as *Xrcc6*, *Ogg1*, *Pole*, and *Neil3* are labeled. **(D)** Gene Ontology enrichment analysis of DEGs. The top 10 significantly enriched biological processes are shown, primarily related to DNA repair, stress response, and double-strand break repair. **(E)** Circular GO plot categorizing enriched GO terms by function: molecular function, cellular component, and biological process. The plot illustrates the number of genes, enrichment level (Rich Factor), and statistical significance (-log10 Q-value) for each term. Created in BioRender. He, Y. (2025) https://BioRender.com/k32w6og.

Six-week-old C57BL/6 male mice were obtained from SLAC Experimental Animal (Shanghai, China) and were housed at 22–24 °C in a 12 h/12 h light/dark cycle with unrestricted access to a standard diet and tap water. After acclimatization feeding, the eight-week-old male mice were randomly divided into four groups: Sham control (Sham), Fisetin treatment (Fisetin), I/R model (I/R), and I/R + Fisetin (I/R+Fisetin). One week before establishing the myocardial I/R injury model, oral gavage was used to provide 40 mg kg-1/day of Fisetin (B20953 Shanghai yuanye Bio-Technology Co., Ltd. Purity: 98%) until the experiment’s terminus. The chemical structure of Fisetin was shown in **Figure 1**. At the end of the experimental procedure, following echocardiography, mice were euthanized under avertin anesthesia (Meilun, China). Heart tissues and serum were collected for analysis.

#### 2.5.2 Establishment of mouse myocardial I/R injury model

Adult male C57BL/6 mice were weighed and anesthetized by intraperitoneal injection of avertin (200 mg/kg). After tracheal intubation, mice were connected to a small animal ventilator. The chest skin was disinfected with povidone-iodine, followed by thoracotomy to expose the heart. The left anterior descending coronary artery (LAD) was ligated with 7–0 suture to induce myocardial ischemia for 45 minutes, followed by reperfusion for 24 hours. Sham-operated mice underwent the same procedure except for the ligation step.

#### 2.5.3 Echocardiography

Mice were anesthetized with avertin (200 mg/kg) and echocardiography was performed using the VINNO D700 high-resolution ultrasound imaging system (VINNO Corporation, Suzhou, China). M-mode echocardiography was used to measure ejection fraction (EF) and fractional shortening (FS). Three consecutive cardiac cycles were recorded for analysis. Successful occlusion of the artery was confirmed by ST- segment elevation on electrocardiogram (ECG). Evaluations were performed 24 hours after I/R injury, with corresponding sham and model groups serving as controls.

#### 2.5.4 TTC staining for infarct size

At 24 hours post-I/R, mice were euthanized by cervical dislocation under anesthesia. Hearts were rapidly excised, rinsed in 0.9% saline, and sectioned. Heart slices were incubated in 2% triphenyltetrazolium chloride (TTC) solution (Solarbio, China) at 37°C in the dark. Viable myocardium stained red, while infarcted areas remained pale. Images were captured using a stereomicroscope (Nikon, Japan), and infarct size was quantified using Image Pro Plus 6.0 software.

#### 2.5.5 Lactate dehydrogenase activity assay

Serum samples were analyzed for LDH activity. LDH levels were measured using a LDH assay kit according to the manufacturer’s instructions (Jiancheng, China). Briefly, 20 μL of sample, 250 μL substrate buffer, and 5 μL coenzyme I added per well and incubated at 37°C for 15 minutes. Then, 250 μL of 2,4-dinitrophenylhydrazine was added, mixed, and incubated for another 15 minutes at 37°C. Next, 250 μL of 0.4 mol/L NaOH was added, mixed, and incubated at room temperature for 5 minutes. Absorbance at 450 nm was measured by microplate reader. LDH activity (U/L) was calculated as:LDH activity = [(OD_sample - OD_control)/(OD_standard - OD_blank)] × standard concentration × dilution factor × 1000.

#### 2.5.6 Creatine kinase-MB content assay

Serum samples were analyzed for CK-MB activity. CK-MB levels were measured using a commercial mouse CK-MB ELISA kit according to the manufacturer’s instructions (JONLNBIO, China). Briefly, 100 μL of standards or samples were added to wells and incubated at 37°C for 90 minutes. Wells were washed, followed by incubation with 100 μL biotinylated antibody at 37°C for 60 minutes. After washing, 100 μL HRP-conjugated reagent was added and incubated at 37°C for 30 minutes. After washing again, 90 μL substrate solution was added and incubated for 15 minutes at 37°C. Finally, 50 μL stop solution was added, and absorbance was measured at 450 nm. CK-MB concentrations were calculated based on the standard curve.

#### 2.5.7 Western blot analysis

Myocardial tissues were lysed in appropriate lysis buffer, and the lysates were centrifuged at 13500 rpm for 20 minutes at 4°C. Supernatants were collected and stored at −80°C. Protein concentrations were determined using the BCA assay. Samples containing 70 μg protein were mixed with 5× loading buffer and PBS, boiled at 100°C for 10 minutes, and subjected to SDS-PAGE. Proteins were transferred onto nitrocellulose membranes by wet transfer. After blocking, membranes were then incubated with secondary anti-rabbit or anti-mouse polyclonal antibodies, including rabbit anti-phospho-histone H2A.X antibody (Cat#: R381558, 1:1000, ZEN-BIOSCIENCE, Chengdu, China), rabbit KU70,Xrcc6 polyclonal antibody (Cat#: 10723-1-AP, 1:1000, Proteintech, Wuhan, China), rabbit β-actin antibody(Cat#: GB15003-100, 1:5000, Servicebio, Wuhan, China) and Goat anti-rabbit IgG H&L (Cat#: 511203, 1:10000, ZEN-BIOSCIENCE, Chengdu, China). After incubation with secondary antibodies, protein bands were detected using ChemiDoc MP system (Bio-Rad Laboratories, USA). β-actin was used as a loading control, and band intensities were quantified for comparison.

#### 2.5.8 ELISA for IL-6 and TNF-α

Serum was collected as above. IL-6 and TNF-α levels were quantified using commercial ELISA kits according to the manufacturer’s protocols (Liankebio, China). Absorbance was measured at 450 nm, and cytokine concentrations were calculated based on standard curves.

#### 2.5.9 Synthesis and transfection of overexpression plasmids and siRNAs

The siRNA of *Xrcc6* was designed and synthesized by GenePharma (Suzhou, Jiangsu, China) (**Tables 1-A, B**). *Xrcc6* plasmids were constructed by HANbio biotechnology (shanghai, China). The pcDNA3.1 (+) empty vector was used as a negative control. Under appropriate conditions, siRNAs and NC were transfected into cells with X-treme GENE siRNA Transfection Reagent (Roche, Mannheim, Germany) and Lipofectamine™ RNAiMAX Transfection Reagent (Thermo Scientific, Carlsbad, USA). Lipofectamine 2000 reagent (Invitrogen, Carlsbad, CA, USA) and ViaFect™ Transfection Reagent (Promega Corporation, Beijing, China) were used to transfect overexpressing plasmids to cells.

**Table 1-A:** The sequences of siRNAs for *Xrcc6*.

siRNA	sense	5’- GGAAGAGAUAGUUUGAUUUTT -3’
	antisense	5’- AAAUCAAACUA UCUCUUCCTG -3’

**Table 1-B:** The sequences of negative control for siRNA.

NC	sense	5’- UUCUCCGAACGUGUCACGUTT -3’
	antisense	5’- ACGUGACACGUUCGGAGAATT -3’

#### 2.5.10 Detection of cellular ROS production

Cellular ROS of cultured cardiomyocytes was examined by fluorometric intracellular ROS Kit (Beyotime, Shanghai, China) according to the manufacturer’s instructions. The samples were observed under a fluorescence microscope (Zeiss, Jena, Germany).

#### 2.5.11 Statistical analysis

Data are expressed as the mean ± SEM. All data and results were explicated in blinded fashion. GraphPad Prism 8.0 software with *post-hoc* multiple testing correction was used to perform statistical analyses. The normality of data was examined by Shapiro-Wilk test before parametric or non-parametric tests. For comparisons, normally distributed data were analyzed by one-way ANOVA analysis followed by Tukey’s *post-hoc* multi-comparison test (multiple-group analysis). For non-normally distributed data, a multiple-group analysis was performed with Kruskal Wallis test with Dunn’s multiple comparisons. No experiment-wide multiple testing correction was applied; only within-test corrections were made. The representative image was selected from repeated experiments to best represent the mean value. ANOVA analysis (for normally distributed data) and Kruskal Wallis tests (for non-normally distributed data) were used to calculate adjusted P values for comparison of multiple groups.

## 3 Results

### 3.1 Establishment of primary cardiomyocyte I/R injury model and gene expression analysis

In this study, an *in vitro* ischemia-reperfusion (I/R) injury model using neonatal mouse primary cardiomyocytes was established, followed by eukaryotic reference-based transcriptome sequencing (**Figure 1A**). Specifically, cardiac tissues were isolated from wild-type neonatal mice, enzymatically dissociated using appropriate kits, and treated with either PBS (control) or H_22_ (I/R simulation). After 24 hours, samples were subjected to RNA sequencing.

Principal component analysis (PCA) was then performed on the transcriptomic data. The 3D PCA plot (**Figure 1B**) showed that PC1 accounted for 93.7% of the variance, clearly separating the NC (normal control) and I/R groups, indicating distinct gene expression profiles between them.

Subsequent differential expression analysis focused on DNA damage response (DDR)–related gene sets. A volcano plot (**Figure 1C**) illustrated the differential gene expression between groups. Genes such as *Neil3*, *Pole*, *Rpa2*, *Rad51*, and *Rdm1* were significantly upregulated, whereas *Xrcc6*, *Ogg1*, *Ercc5*, *Ercc1*, and *Polk* were downregulated, suggesting that these genes may play critical roles in cardiomyocyte I/R injury.

Furthermore, Gene Ontology (GO) enrichment analysis of the differentially expressed genes (DEGs) was conducted. **Figure 1D** displays the top 10 enriched GO terms, with notable enrichment in DNA repair–related processes. **Figure 1E** presents a circular visualization of enriched terms across different GO categories (molecular function, biological process, cellular component), indicating that alterations in DNA repair–associated gene expression may represent a key molecular mechanism underlying myocardial I/R injury. These findings provide important insights into the potential regulatory pathways involved.

### 3.2 Subtype analysis of cardiomyocytes and identification of key genes in the I/R mouse model

**Figure 2:**
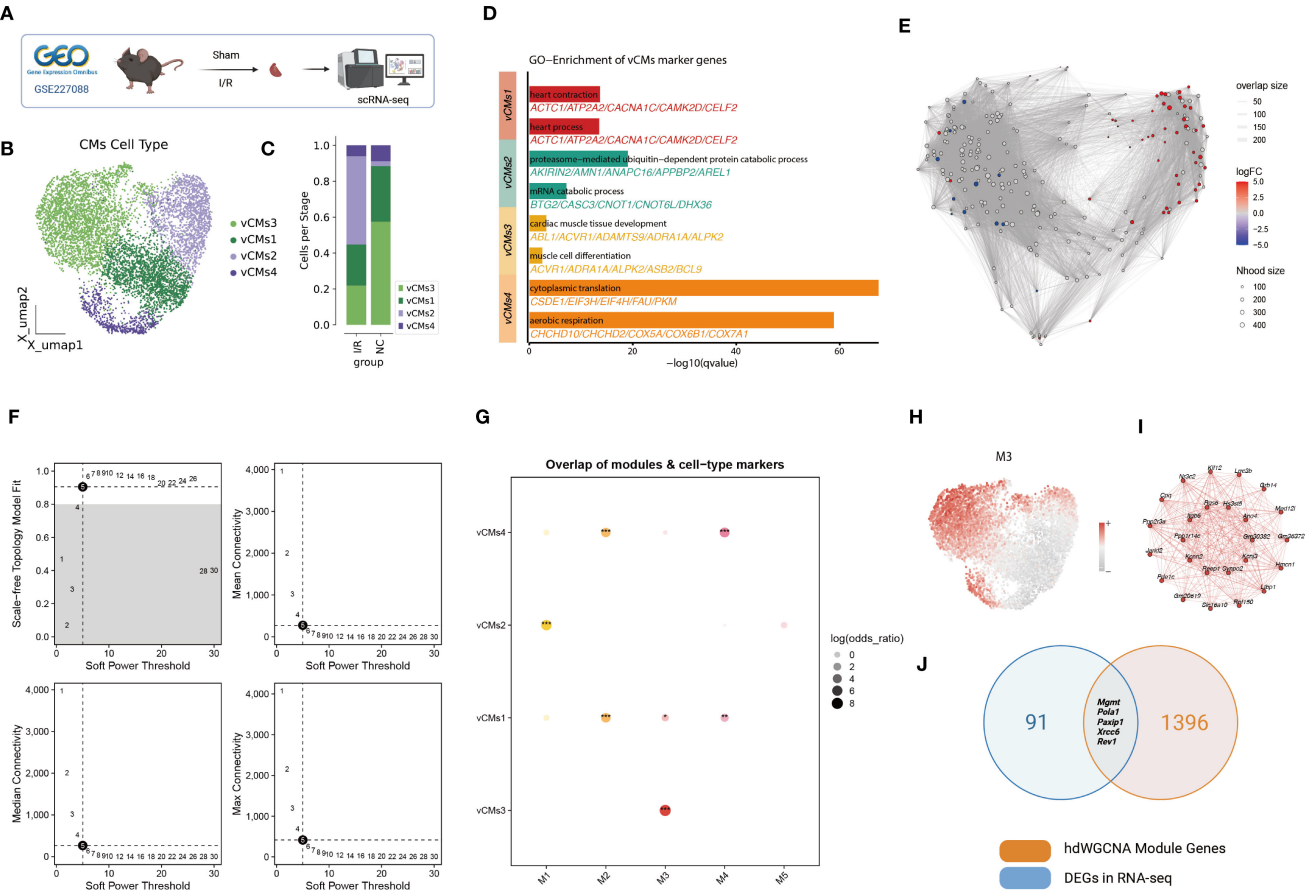
Single-nucleus RNA-seq analysis of the murine myocardial ischemia/reperfusion (I/R) model. **(A)** Schematic of snRNA-seq workflow. Cardiac tissues were collected from Sham and I/R groups for single-nucleus RNA sequencing. **(B)** UMAP clustering of ventricular cardiomyocytes revealed four distinct subtypes (vCMs1–vCMs4). **(C)** Proportional distribution of vCM subtypes across Sham and I/R groups. **(D)** Gene Ontology enrichment analysis of marker genes for each vCM subtype. **(E)** Neighborhood abundance analysis using the *miloR* algorithm showed a significant increase in vCMs2 and a decrease in vCMs3 under I/R conditions. **(F)** Determination of the soft-thresholding power for hdWGCNA network construction. **(G)** Correlation between five hdWGCNA gene modules (M1–M5) and the vCM subtypes. **(H)** UMAP visualization of module M3 activity scores across cardiomyocyte populations. **(I)** Co-expression network of the top 20 hub genes within module M3. **(J)** Overlap between M3 module genes and differentially expressed DNA repair genes identified in bulk RNA-seq analysis.

To explore cardiomyocyte subtype-specific responses in ischemia/reperfusion (I/R) injury, we conducted a comprehensive analysis of single-nucleus RNA-sequencing (snRNA-seq) data from the I/R mouse model (GEO: GSE270888) (**Figure 2A**) (**Supplementary Table 2**). Comparative analysis revealed marked differences in both cellular composition (**Supplementary Figure S1A**) and transcriptomic profiles (**Supplementary Figure S1C**) between Sham and I/R heart tissues. Notably, the proportion of cardiomyocytes was significantly reduced in the I/R group (**Supplementary Figure S1B**), consistent with the pathological features of I/R injury. Ischemia and hypoxia trigger cardiomyocyte apoptosis and necrosis, leading to a loss of functional myocardium. This depletion of cardiomyocytes impairs cardiac contractility and underlies many I/R-related pathophysiological manifestations, including heart failure.

Prior to clustering, batch effects across replicates were corrected using Harmony, which effectively minimized technical variation while preserving biologically meaningful heterogeneity (**Supplementary Figure S2**).

Given this, we focused on cardiomyocyte subpopulations and their functional roles. Dimensionality reduction and unsupervised clustering identified four distinct ventricular cardiomyocyte subtypes (vCMs1–vCMs4) (**Figure 2B**). Comparative analysis revealed that vCMs2 was significantly enriched, while vCMs3 was markedly reduced in I/R hearts (**Figure 2C**). These shifts were validated by local neighborhood differential abundance analysis using the *miloR* algorithm, which confirmed a significant increase in vCMs2 and a decrease in vCMs3 under I/R conditions (**Figure 2E**).

Gene Ontology (GO) enrichment of subtype-specific marker genes revealed distinct functional signatures (**Figure 2D**). vCMs1 was enriched for pathways related to heart contraction and cardiac processes, indicating its role in maintaining normal cardiac function. vCMs2 showed enrichment in proteasome-mediated ubiquitin-dependent protein catabolism and mRNA catabolic processes, suggesting a pathological phenotype associated with post-I/R stress responses. vCMs3 was associated with cardiac muscle tissue development and muscle cell differentiation, implying a role in tissue regeneration and repair. vCMs4 was enriched in cytoplasmic translation and aerobic respiration pathways, indicating a function in energy metabolism and protein synthesis.

To elucidate regulatory mechanisms underlying these subtypes, we applied the high-dimensional Weighted Gene Co-expression Network Analysis (hdWGCNA) algorithm to construct a gene co-expression network (**Figure 2F**; **Supplementary Figures S1D–G**). Five distinct modules (M1–M5) were identified, among which module M3 showed strong correlation with vCMs3 (**Figure 2G**). Visualization of module activity scores in the UMAP space confirmed that M3 was predominantly active in vCMs3 (**Figure 2H**), suggesting that it may define the molecular signature of this regenerative cardiomyocyte subtype. The top 20 hub genes in M3 formed a tightly interconnected network, further underscoring the module’s regulatory relevance (**Figure 2I**).

To identify candidate genes involved in I/R injury and repair, we intersected M3 module genes with differentially expressed DNA repair genes from our bulk RNA-seq analysis. This integrative approach yielded five potential key regulators: *Mgmt*, *Pola1*, *Paxip1*, *Xrcc6*, and *Rev1* (**Figure 2J**). These genes may play critical roles in mediating DNA damage response and repair in vCMs3 cells, offering promising targets for mechanistic studies and therapeutic intervention in myocardial I/R injury.

### 3.3 Pseudotime trajectory analysis of cardiomyocyte subtypes and identification of cell fate–associated genes

**Figure 3:**
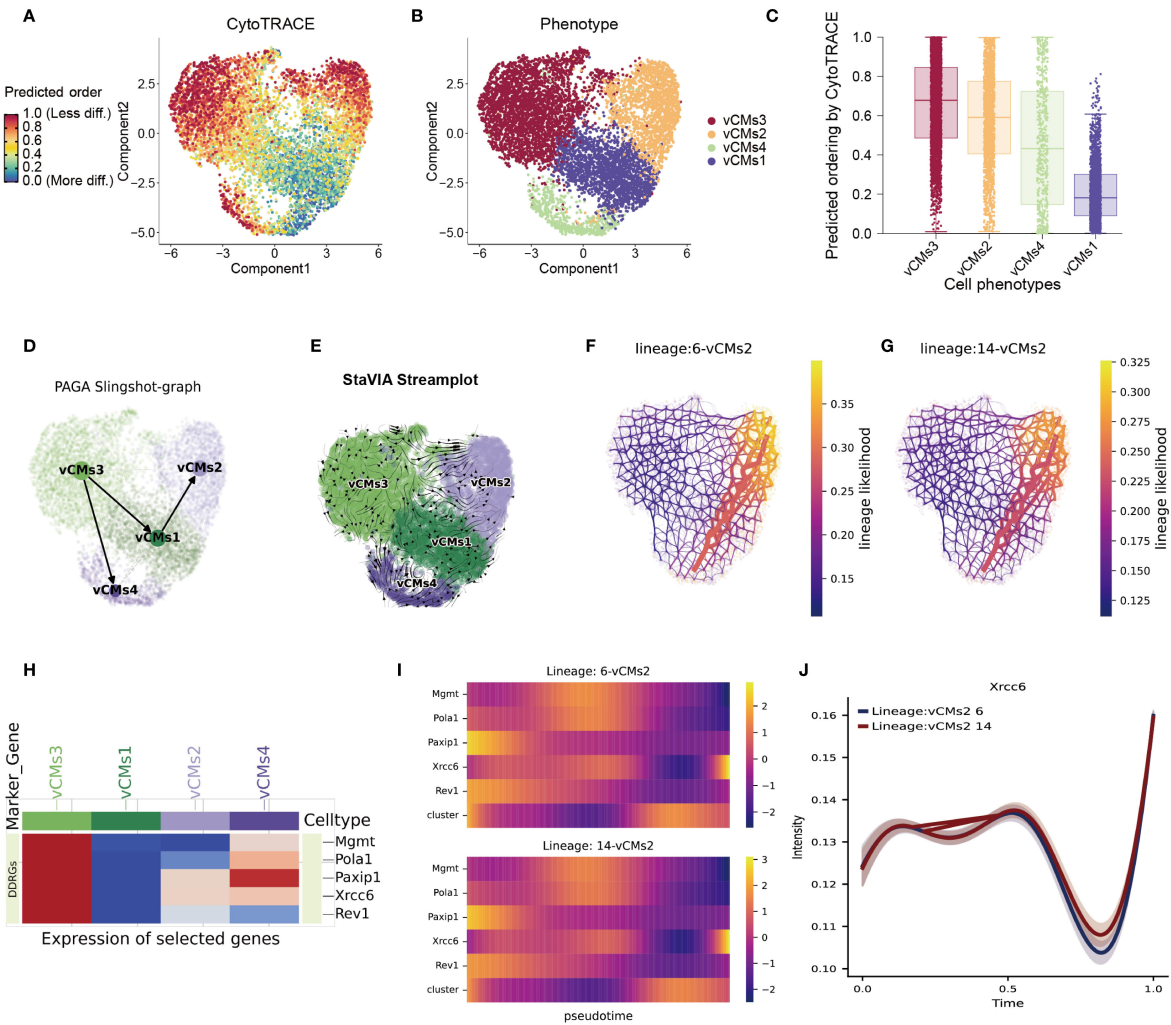
Integrated trajectory inference reveals cardiomyocyte differentiation pathways and key regulatory factors. **(A–C)** Predicted differentiation potential of cardiomyocyte subtypes as assessed by the CytoTRACE2 algorithm, highlighting vCMs3 as the most undifferentiated population. **(D)** Developmental trajectories among cardiomyocyte subtypes inferred using the Slingshot algorithm, visualized via a PAGA graph. **(E)** Differentiation trajectories reconstructed with high resolution using the VIA algorithm, confirming two major branching paths. **(F–G)** Two principal lineage branches identified by VIA, illustrating distinct differentiation directions within the cardiomyocyte population. **(H)** Heatmap showing the expression profiles of candidate regulatory genes across the four vCM subtypes. Several genes display subtype-specific expression, including *Xrcc6*, which is markedly upregulated in vCMs3. **(I)** Dynamic expression patterns of key regulatory genes along the two differentiation trajectories, revealing their potential roles in cardiomyocyte fate determination. **(J)** Pseudotime expression profile of *Xrcc6*, indicating its biphasic regulation during cardiomyocyte differentiation.

To systematically elucidate the mechanisms underlying cardiomyocyte fate decisions during differentiation, we employed and compared three trajectory inference algorithms: Slingshot, Palantir, and StaVIA. CytoTRACE2 analysis first revealed that the vCMs3 population exhibited the highest developmental potential, suggesting that these cells represent an early, less differentiated state and may play a critical role in myocardial repair following injury (**Figures 3A–C**). Trajectory reconstruction using the Slingshot algorithm clearly delineated two major developmental branches originating from vCMs3, diverging toward vCMs2 and vCMs4, respectively (**Figure 3D**; **Supplementary Figure S3A**). This bifurcated structure was further validated by Palantir’s PAGA graph, which demonstrated consistent pseudotime progression and differentiation directionality from vCMs3 toward more mature subtypes (**Supplementary Figure S3B**). The StaVIA algorithm further refined the topological structure of the trajectories with high resolution, confirming the two primary differentiation paths and their sequential temporal transitions (**Figure 3E**; **Supplementary Figure S3C**).

Focusing on the two major differentiation branches identified by VIA (**Figures 3F–G**), we next analyzed the expression patterns of candidate regulatory genes across the four main cardiomyocyte subtypes (vCMs1–4). Heatmap visualization revealed that many candidate genes displayed subtype-specific expression patterns—for example, *Xrcc6* was highly expressed in vCMs3 but significantly downregulated in vCMs2—suggesting distinct regulatory roles in divergent differentiation trajectories (**Figure 3H**). Dynamic gene expression analysis along both differentiation branches showed a biphasic expression pattern of *Xrcc6*, characterized by an initial decline followed by reactivation (**Figures 3I, J**). This expression trend suggests that *Xrcc6* may serve as a key regulator in cardiomyocyte fate determination, facilitating lineage commitment toward distinct functional subtypes.

In summary, our study uncovers substantial heterogeneity among cardiomyocyte subpopulations and delineates critical fate bifurcations during myocardial differentiation in the context of disease. Notably, vCMs3 cells exhibit high developmental plasticity and undergo marked trajectory shifts and functional reprogramming under pathological conditions. *Xrcc6* emerges as a potential master regulator in the transition from a progenitor-like state to disease-responsive cardiomyocyte subtypes. These findings provide new mechanistic insights into cardiomyocyte fate specification and offer potential avenues for therapeutic intervention and target development in cardiac regeneration and disease.

### 3.4 *Xrcc6* regulates macrophage polarization following myocardial ischemia–reperfusion injury

**Figure 4:**
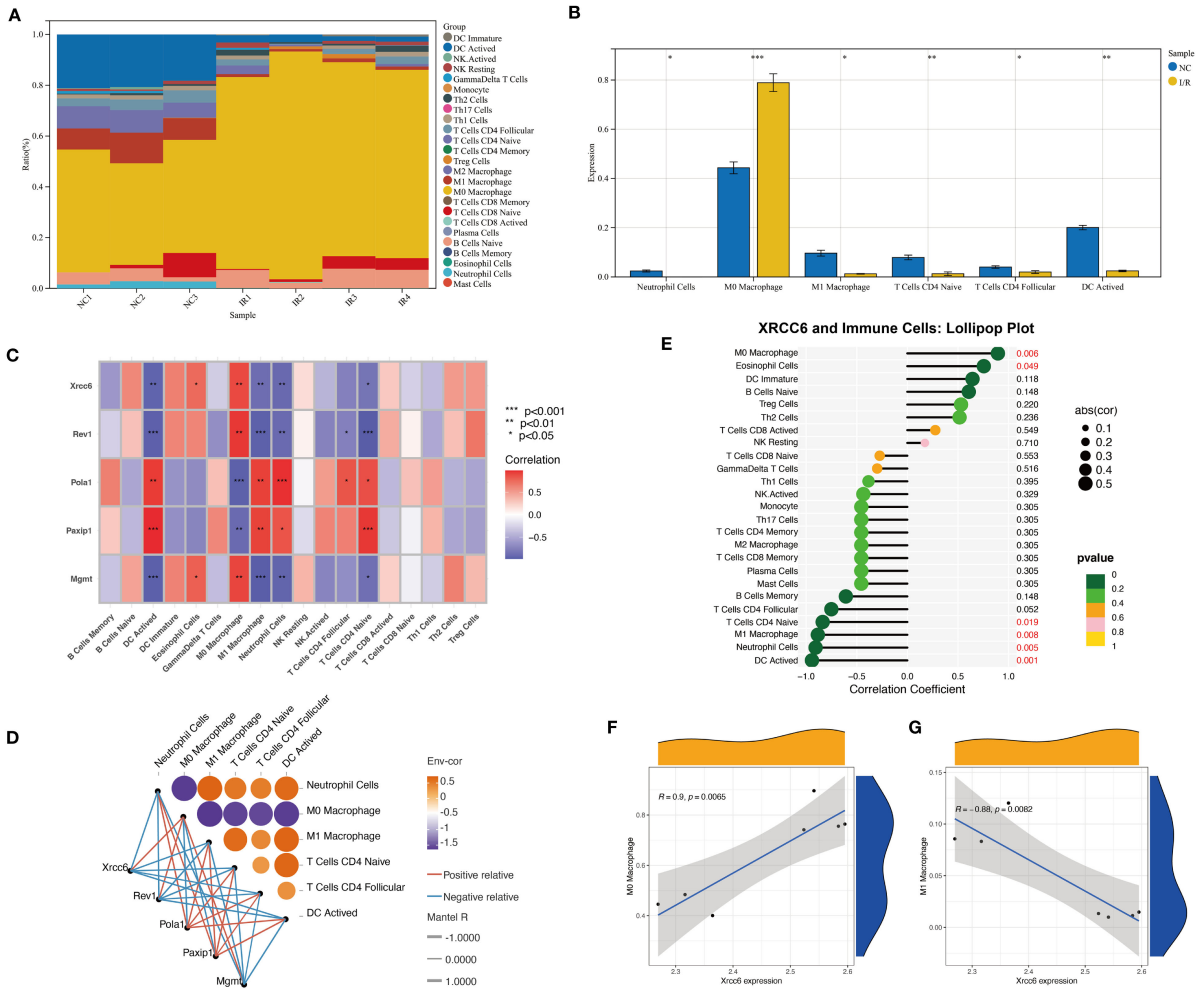
Xrcc6 links DNA repair to immune cell heterogeneity in ischemia-reperfusion. **(A)** Stacked bar plot showing the relative proportions of different immune cell subtypes in each sample. **(B)** Differential analysis of major immune cell subtypes between the Sham and I/R groups. **(C)** Heatmap of the correlations between Xrcc6 and associated DNA repair genes across different immune cell types. **(D)** Correlation network diagram illustrating the relationships between Xrcc6, its related genes, and immune cell subpopulations. **(E)** Lollipop plot showing the correlation between Xrcc6 expression and immune cell types, annotated with p-values and correlation coefficients. **(F–G)** Scatter plots with fitting lines illustrating the relationship between Xrcc6 expression levels and the proportion of M0/M1 macrophages, accompanied by Pearson correlation coefficients and significance annotations.

To investigate the dynamic changes in immune cell composition during myocardial injury and repair, and to elucidate the role of the key regulatory factor *Xrcc6*, we employed the CIBERSORT algorithm to compare the proportions of immune cell subtypes between the Sham and ischemia–reperfusion (I/R) groups (**Supplementary Table 3**). The analysis revealed that I/R significantly altered immune cell composition, notably characterized by an increased proportion of M0 macrophages and a marked decrease in M1 macrophages (**Figures 4A, B**). This shift suggests a transition of the immune microenvironment toward a reparative phenotype. In addition to macrophages, several T cell subtypes and dendritic cells also exhibited varying degrees of compositional changes, reflecting a broad spectrum of immune remodeling.

To further characterize the expression of regulatory genes across immune cell subtypes, we constructed a correlation heatmap between DNA repair-related genes and immune cell populations. Several genes displayed strong positive correlations with multiple immune cell types. Notably, *Xrcc6* exhibited distinct expression patterns between M0 and M1 macrophages (**Figure 4C**). Both Mantel testing and bivariate correlation analyses confirmed a significant positive correlation between *Xrcc6* expression and M0 macrophages, and a negative correlation with M1 macrophages (**Figure 4D**). These findings suggest that *Xrcc6* may play a critical role in mediating the transition from a pro-inflammatory to a reparative macrophage phenotype. This conclusion was further supported by lollipop and scatter plots, where *Xrcc6* expression showed a strong positive correlation with M0 macrophages (r = 0.69, p < 0.01) and a strong negative correlation with M1 macrophages (r = –0.88, p < 0.001) (**Figures 4E–G**).

**Figure 5:**
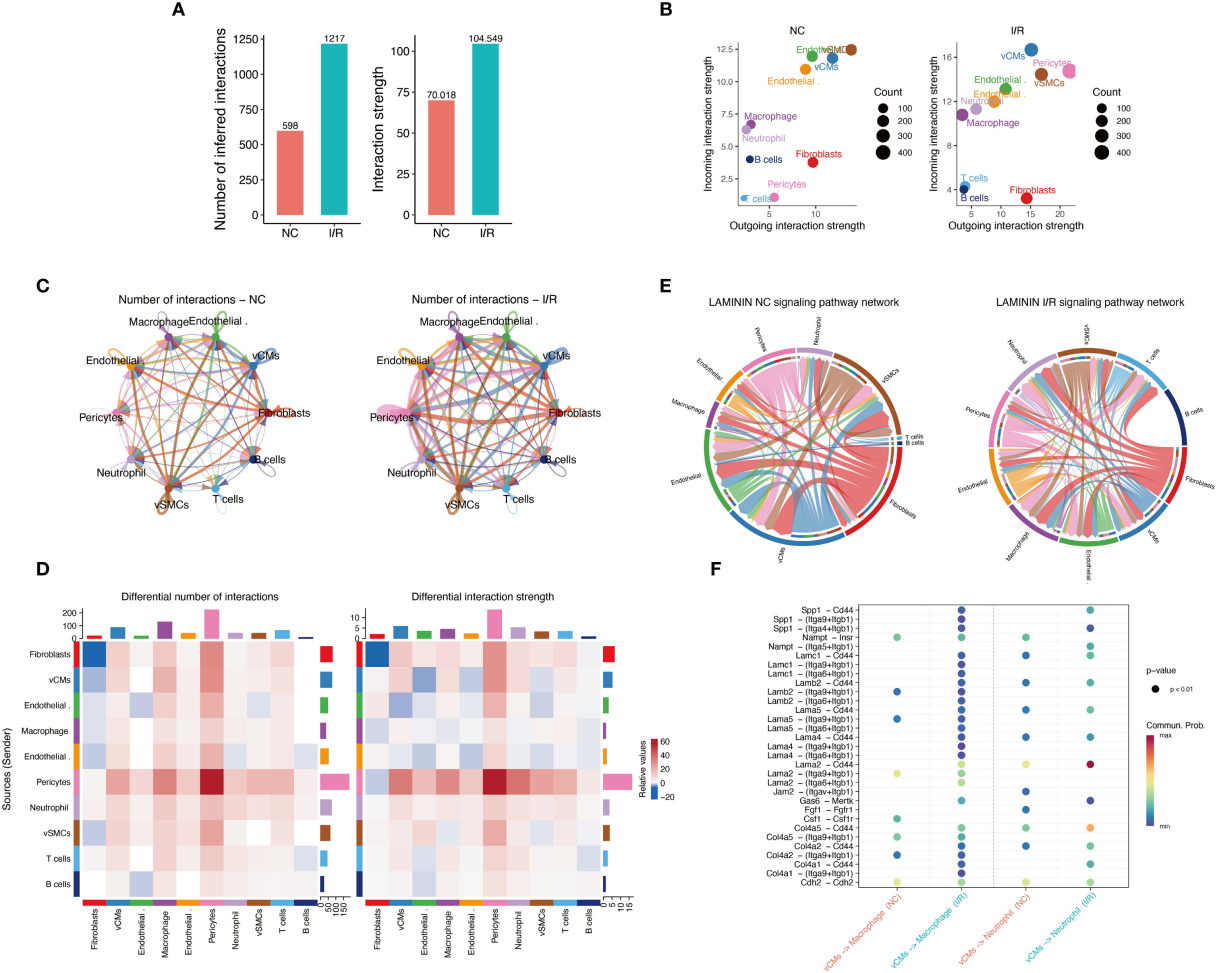
Characterization of intercellular communication networks before and after I/R. **(A)** Comparison of the total number of predicted intercellular communication events and the overall interaction strength. **(B)** Analysis of communication output (Outgoing) and input (Incoming) intensities across different cell types: The left panel represents the Sham group, and the right panel represents the I/R group. The size of the points indicates the number of communication events (Count), with colors differentiating cell types. **(C)** Intercellular communication network diagrams: The left panel shows the Sham group, and the right panel shows the I/R group. Line thickness indicates the interaction strength, and color represents the source cell type. **(D)** Differential interaction heatmap: The left panel illustrates the difference in the number of intercellular communication events between the Sham and I/R groups, while the right panel shows differences in communication strength. The intensity of the color reflects the magnitude of change, with red indicating enhanced communication in the I/R group and blue indicating reduced communication. **(E)** Laminin signaling pathway network circular diagram: Depicts the direction of signal flow and participating cell types in the Laminin pathway in the Sham (left) and I/R (right) groups. **(F)** Significance bubble plot of key ligand-receptor pairs in the Laminin pathway: Displays the communication probability (color) and significance (p-value) differences for various ligand-receptor pairs between the Sham and I/R groups.

To assess the overall impact of I/R on intercellular communication, we quantified the number and intensity of cell–cell interactions in both groups (**Supplementary Figure S3D**). The I/R group exhibited significantly increased cellular interactions, with the number of inferred interactions rising from 598 to 1,217 and the total interaction strength increasing from 70.018 to 104.549 (**Figure 5A**), indicating a more active and robust intercellular communication network. Cardiomyocytes, smooth muscle cells, and macrophages in the I/R group showed particularly enhanced signaling input and output (**Figure 5B**). Network topology analysis further revealed that I/R substantially strengthened interactions among these three cell types, leading to a more complex interaction network (**Figures 5C, D**). At the signaling pathway level, increased activation of key axes such as Laminin was observed in the I/R group, suggesting this pathway may mediate critical intercellular signaling events involved in immune remodeling and tissue repair (**Figures 5E, F**).

In summary, *Xrcc6* may promote immune microenvironment remodeling following myocardial ischemia–reperfusion injury by regulating macrophage polarization. The opposing correlation patterns of *Xrcc6* with M0 and M1 macrophages, together with enhanced intercellular communication and activation of specific signaling pathways such as Laminin, suggest that *Xrcc6* functions during the early stages of tissue injury to modulate immune cell phenotypic transitions. These findings provide novel insights into immune regulation in myocardial injury and highlight *Xrcc6* as a potential target for therapeutic intervention.

### 3.5 Molecular docking and stability validation of Fisetin targeting *Xrcc6*

This study employed molecular docking and molecular dynamics simulation techniques to analyze the interaction between Fisetin and *Xrcc6* in detail, revealing the stability, interaction mechanisms, and potential role of this binding in cardiac injury repair. The simulation results offer insights into the stability, interaction patterns, and biological implications of the quercetin-*Xrcc6* complex from multiple perspectives.

#### 3.5.1 Molecular docking

**Figure 6:**
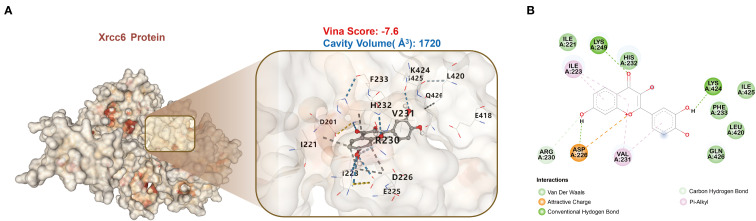
Molecular docking analysis reveals high-affinity binding of Fisetin to Xrcc6. **(A)** Molecular docking results of Fisetin with the *Xrcc6* protein. The Vina score of -7.6 and the binding site cavity volume of 1720 Å³ indicate a high binding stability between Fisetin and *Xrcc6*. **(B)** Schematic diagram illustrating the specific interactions between Fisetin and *Xrcc6*. Fisetin binds with several amino acid residues through various types of interactions, including hydrogen bonds, van der Waals forces, electrostatic attraction, and π-alkyl interactions.

The molecular docking analysis indicated a strong interaction between Fisetin and the *Xrcc6* protein (**Figure 6A**). Analysis of the protein’s three-dimensional structure showed that Fisetin binds to the binding site of *Xrcc6*, establishing tight interactions with several amino acid residues via hydrogen bonds, van der Waals forces, electrostatic interactions, and π-alkyl interactions. The Vina score of -7.6 suggests that the quercetin-*Xrcc6* binding is highly stable. Moreover, the binding site’s cavity volume of 1720 Å³ further supports the adaptability and stability of Fisetin binding. Key interactions include hydrogen bonds with residues LYS-249, LYS-424, and ARG-230; van der Waals interactions with ILE-221, HIS-232, ILE-425, PHE-223, LEU-420, and GLN-426; electrostatic interaction with ASP-226; and π-alkyl interactions with ILE-223 and VAL-231 (**Figure 6B**). These multiple interactions indicate that quercetin’s binding to the *Xrcc6* protein is mediated through various modes, demonstrating a strong binding capacity.

#### 3.5.2 Conformational stability of the complex

**Figure 7:**
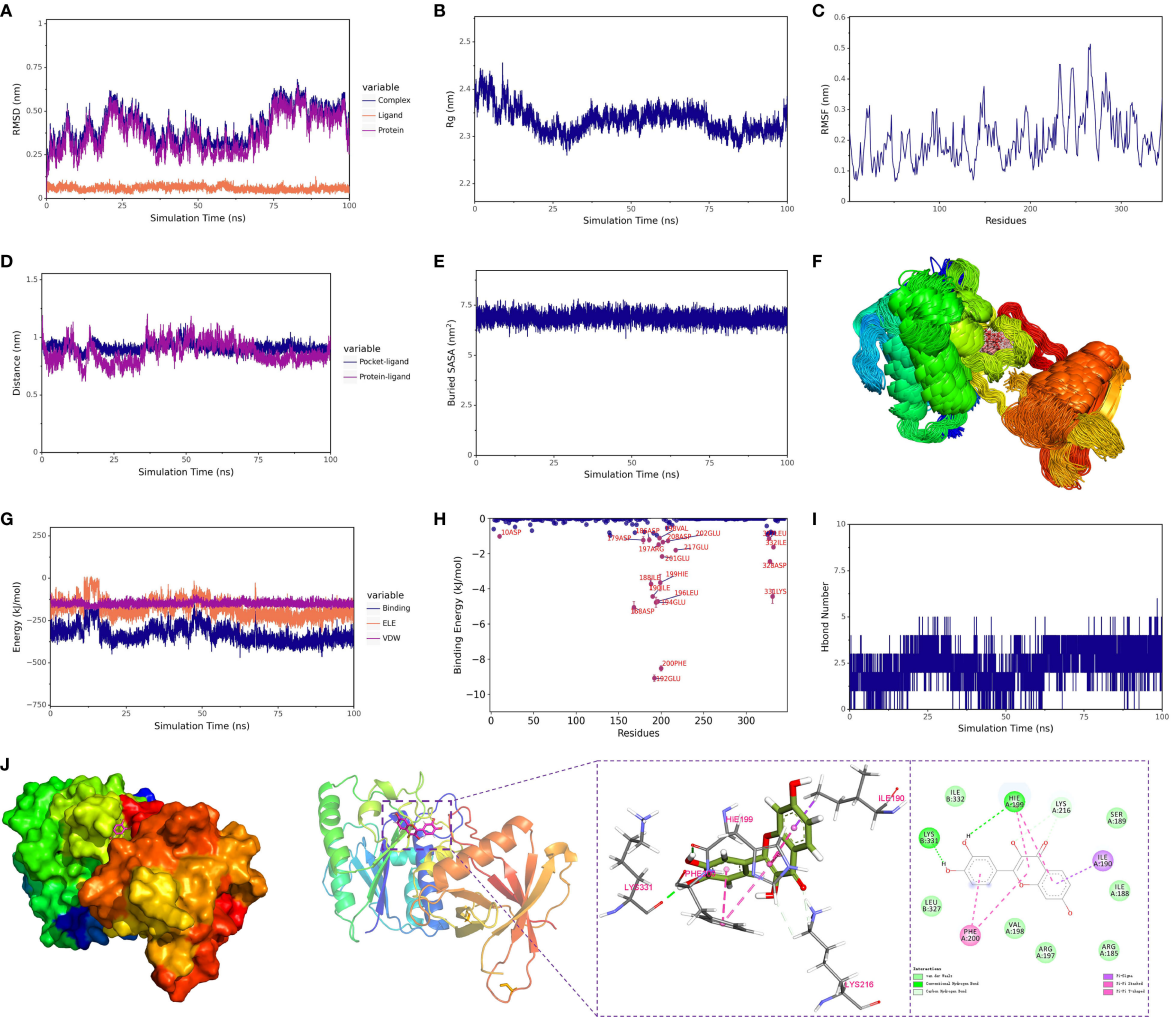
Structural dynamics and energetic analysis of protein-ligand binding via molecular dynamics simulation. **(A)** RMSD of the complex, protein, and small molecule ligand. **(B)** Radius of gyration (Rg) of the complex. **(C)** RMSF (Root Mean Square Fluctuation) of the protein within the complex. **(D)** Distance between the protein and the small molecule binding site (Dock site-ligand). **(E)** Buried solvent-accessible surface area (Buried SASA) between the small molecule and the protein. **(F)** Superimposition of simulation conformations. **(G)** Binding energy (VDW and ELE) between the small molecule and protein. **(H)** Contribution of amino acids to the binding energy. **(I)** Number of hydrogen bonds (Hbond number). **(J)** Interactions between the protein and small molecule. ***1 kcal/mol = 4.184 kJ/mol***.

A 100 ns molecular dynamics simulation was conducted to assess the overall stability of the quercetin–*Xrcc6* complex. RMSD (Root Mean Square Deviation) analysis revealed slight fluctuations in the complex’s structure at the beginning of the simulation, followed by stabilization (**Figure 7A**). The RMSD of the protein backbone mirrored that of the overall complex, indicating that the binding of the small molecule did not significantly disrupt the protein’s conformation, and the binding state remained stable throughout the simulation. Additionally, Radius of Gyration (Rg) analysis showed that the complex maintained a compact and stable conformation during the entire simulation period (**Figure 7B**), suggesting no significant structural relaxation or denaturation of the protein. A modest RMSD increase after ~75 ns was attributed to the intrinsic flexibility of loop and terminal regions distal to the binding pocket. Importantly, RMSF analysis confirmed that residues in the binding pocket exhibited minimal fluctuations, indicating that ligand binding remained stable despite global backbone breathing motions.

#### 3.5.3 Local flexibility and dynamic behavior

RMSF (Root Mean Square Fluctuation) analysis was performed to evaluate the flexibility of each residue in the protein (**Figure 7C**). Results showed minimal fluctuations around the binding site residues (e.g., LYS-331, HIE-199, PHE-200), indicating a stable conformation in this region, which may serve as the critical core for binding. Principal Component Analysis (PCA) further supported this, revealing that the conformational distribution of the small molecule was restricted to a few principal components, signifying high conformational stability in the bound state.

#### 3.5.4 Binding site and spatial behavior analysis

**Figure 8:**
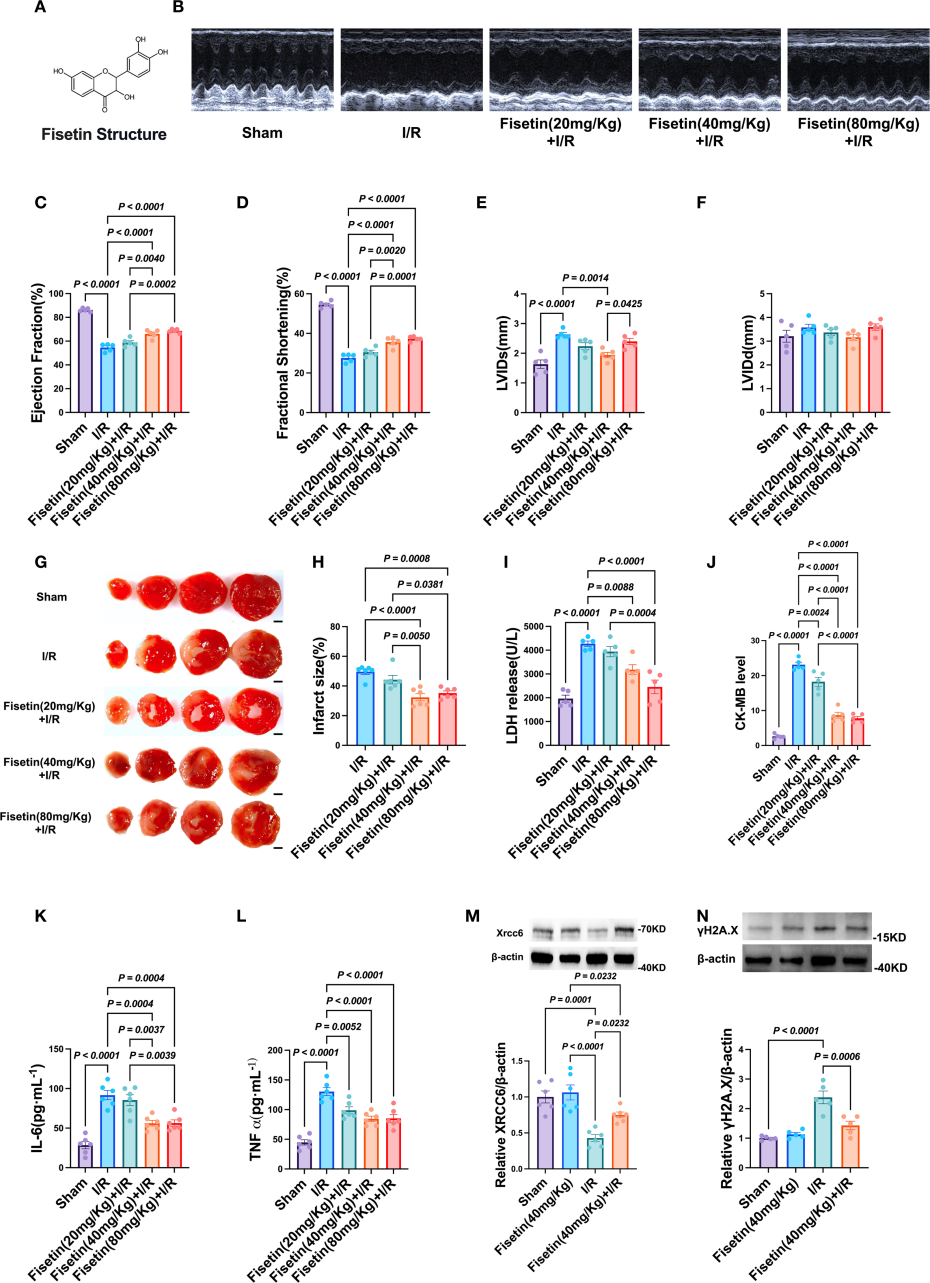
Protective effects of Fisetin on myocardial ischemia-reperfusion injury in mice. **(A)** Chemical structure of Fisetin (FIS). **(B–F)** Cardiac function evaluated by echocardiography in Sham group, I/R group, 20 mg/Kg FIS treatment group, 40 mg/Kg FIS treatment group and 80 mg/Kg FIS treatment group. (n=5) **(G, H)** Representative images of TTC staining and infarct size in FIS treatment mice after I/R injury. Scale bar = 2 mm. (white represents infarcted tissue, red represents viable myocardium) (n=6). **(I, J)** Serum levels of LDH and CK-MB in each group (n=5). **(K, L)** ELISA analysis measuring levels of inflammatory cytokines IL-6 and TNF-α (n=6). **(M, N)** Western blot analysis of *Xrcc6* and γH2A.X protein expression levels in myocardial tissue (n=5-6). Created in BioRender. He, Y. (2025) https://BioRender.com/hrbcp0z.

The spatial distance and binding mode between the small molecule and the protein were analyzed using centroid distance evolution and Buried SASA (Solvent Accessible Surface Area) analysis. The distance between the binding site residues and the small molecule’s centroid fluctuated slightly at the beginning of the simulation but rapidly stabilized thereafter (**Figure 8D**). Simultaneously, the Buried SASA value also reached stability (**Figure 7E**), reflecting the progressive embedding of the small molecule into the binding site, forming a stable buried structure with the protein. Additionally, the superposition of simulated conformations (**Figure 7F**) confirmed that the small molecule remained bound to the specific site without significant dissociation or repositioning.

#### 3.5.5 Molecular interaction mechanisms

Hydrogen bond analysis showed that approximately 1–4 hydrogen bonds were maintained between the small molecule and *Xrcc6* throughout the simulation (**Figure 7I**). Frequency analysis revealed that residues such as LYS-331 had a high occupancy rate for hydrogen bonding with the small molecule (up to 83%) (**Table 2**), indicating their significant role in maintaining binding stability. Furthermore, non-covalent interactions, including hydrophobic interactions (e.g., ILE-190, PHE-200, HIE-199) and π-π stacking interactions (**Figure 7J**), were also present, contributing to the overall structural stability of the complex.

**Table 2:** Hydrogen bond frequency between small molecule and protein.

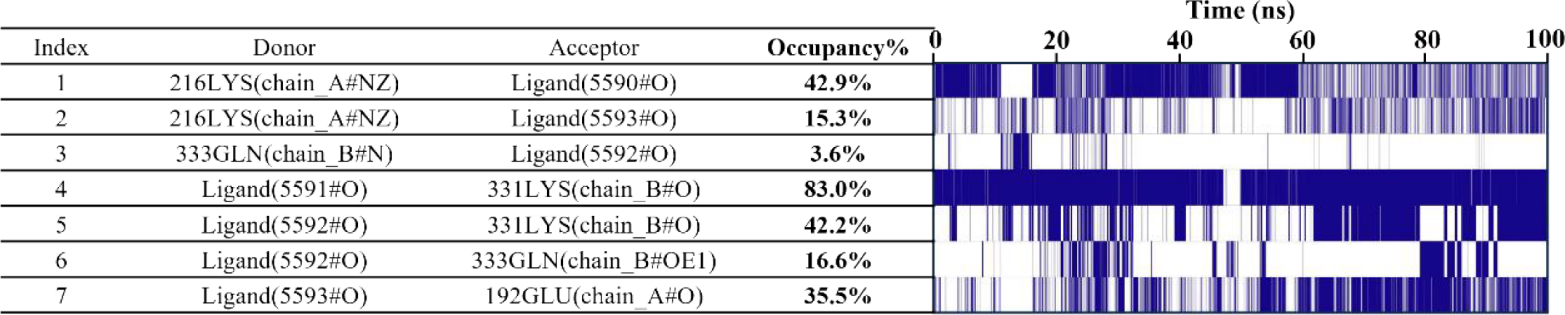

#### 3.5.6 Binding free energy analysis

The dynamic changes in electrostatic (ELE) and van der Waals (VDW) interaction energies indicated stabilization in the latter part of the simulation (**Figure 7G**), suggesting that the interaction between the small molecule and protein reached equilibrium. Binding free energy calculations based on the MM-PBSA method (**Table 3**) showed that the total free energy of the complex (ΔE_MMPBSA) was –7.55 ± 0.59 kcal/mol. Van der Waals interactions (ΔE_vdw = –38.85 ± 0.16 kcal/mol) were the primary contributors, followed by electrostatic interactions (ΔE_ele = –23.13 ± 0.42 kcal/mol), with hydrophobic interactions (ΔE_nonpol = –4.25 ± 0.01 kcal/mol) as a secondary factor. These results indicate that the binding of fisetin to Xrcc6 is mainly driven by hydrophobic and van der Waals forces, with electrostatic interactions playing an auxiliary role in stabilizing the complex structure.

**Table 3:** Binding energy and its components at steady state (units: kcal/mol).

Complex	ΔEvdw	ΔEele	ΔEpol	ΔEnonpol	ΔEMMPBSA	−TΔS	ΔGbind*
Protein-Ligand	–38.843 ± 0.161	-23.129 ± 0.420	58.672 ± 0.839	–4.255 ± 0.007	–7.554 ± 0.594	5.775 ± 0.621	–1.780 ± 0.704

*ΔGbind=ΔEvdw+ΔEele+ΔEpol+ΔEnonpol−TΔS .

#### 3.5.7 Identification of key residues

The binding energy decomposition analysis identified key residues that significantly contribute to the binding of the small molecule (**Supplementary Table 4**), including GLU-192, PHE-200, and LYS-331 (**Figure 7H**). These residues form multiple non-covalent interactions with the small molecule, constituting the core region of the binding pocket, suggesting their potential role as structural-functional hotspots.

In conclusion, this study systematically analyzed the binding stability and interaction mechanisms of Fisetin with *Xrcc6* through 100 ns molecular dynamics simulations. The results indicated that Fisetin stably binds to a specific site on the *Xrcc6* protein, with van der Waals forces predominantly driving the binding, complemented by electrostatic and hydrophobic interactions that collectively maintain the structural stability of the complex. Binding free energy calculations and key residue identification further highlighted the critical role of PHE-200, GLU-192, and LYS-331 in molecular recognition and binding. These findings provide theoretical and structural foundations for understanding the mechanism of Fisetin and its potential as a regulator of *Xrcc6*.

#### 3.5.8 Fisetin attenuates myocardial ischemia–reperfusion injury in mice

To evaluate the protective effects of Fisetin against myocardial ischemia–reperfusion (I/R) injury, we pretreated 6–8-week-old male C57BL/6J mice with Fisetin at different doses (20, 40, or 80 mg/kg) for one week prior to I/R induction. Cardiac function was subsequently assessed by echocardiography. As expected, I/R significantly impaired cardiac function; however, mice pretreated with 40 or 80 mg/kg Fisetin exhibited marked improvement, with no significant differences observed between these two groups (**Figures 8B–F**). Myocardial infarct size, determined by TTC staining, was significantly reduced in the 40 and 80 mg/kg groups, again with no detectable differences between them (**Figures 8G, H**). Consistently, serum levels of the myocardial injury markers LDH and CK-MB were markedly elevated in the I/R group but significantly decreased in both the 40 and 80 mg/kg Fisetin groups (**Figures 8I, J**). ELISA analysis further revealed that serum levels of the pro-inflammatory cytokines TNF-α and IL-6 were significantly lower in the Fisetin-pretreated groups compared with the I/R group, with no significant differences between the 40 and 80 mg/kg groups (**Figures 8K, L**). Collectively, these results demonstrate that Fisetin substantially attenuates myocardial I/R injury in mice.

To further elucidate the underlying mechanism, we examined the expression of two key proteins involved in the repair of DNA double-strand breaks (DSBs): *Xrcc6* and γH2A.X. *Xrcc6* plays a central role in promoting DSB repair, whereas γH2A.X serves as a marker of DSB damage. I/R markedly downregulated *Xrcc6* expression and upregulated γH2A.X expression. Notably, pretreatment with 40 mg/kg Fisetin effectively restored the expression of both proteins (**Figures 8M, N**). These findings suggest that Fisetin may alleviate myocardial I/R injury, at least in part, by enhancing DSB repair through the modulation of *Xrcc6* and γH2A.X expression.

#### 3.5.9 *Xrcc6* plays a critical role in H_22_-induced cardiomyocyte injury

**Figure 9:**
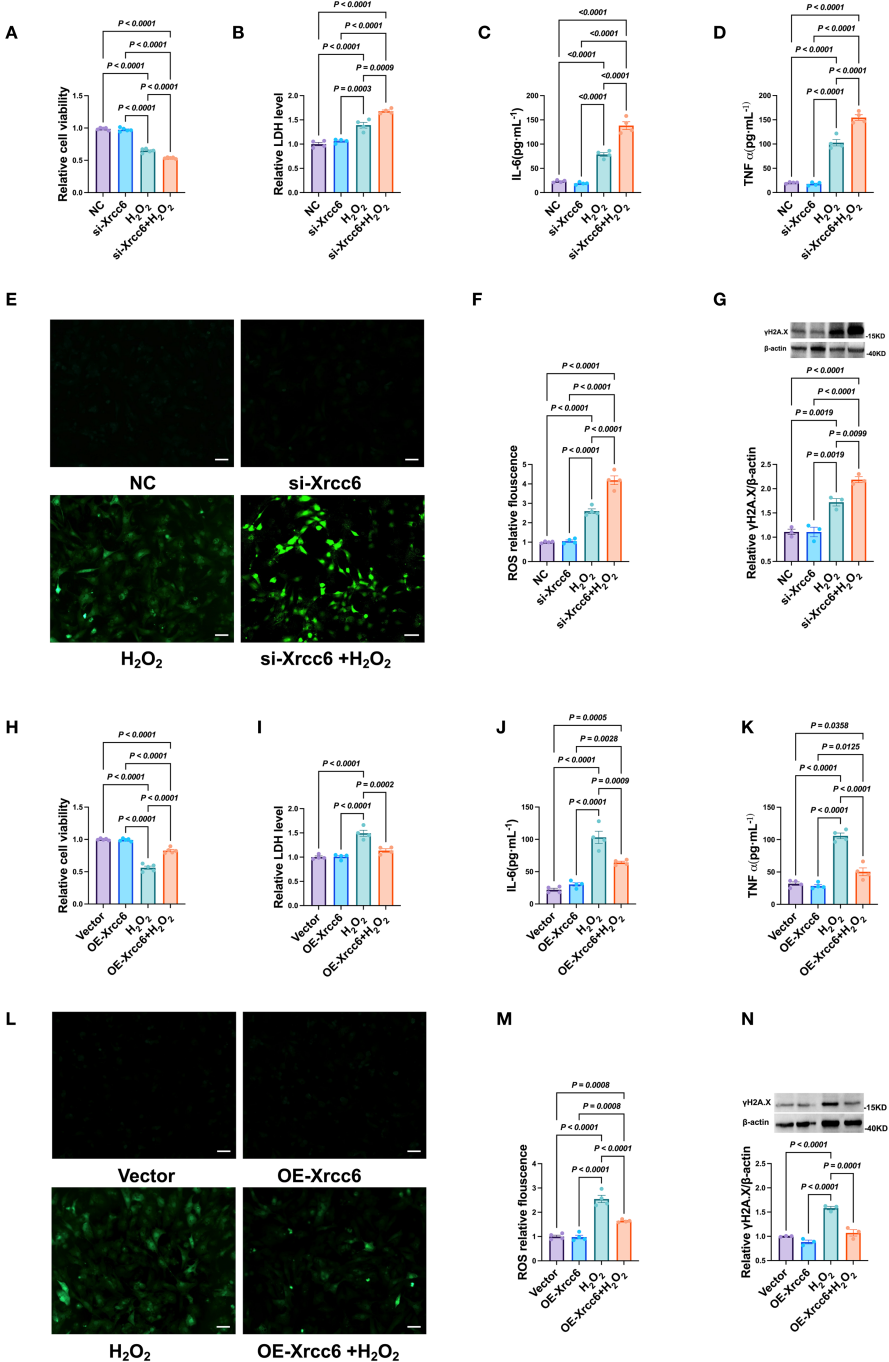
Effects of Xrcc6 on cardiomyocytes injury induced by H2O2. **(A)** Cell viability after transfection of si *Xrcc6*. n = 4. **(B)** The activity of LDH after transfection of si *Xrcc6*. n = 4. **(C, D)** Normalized IL-6 and TNF-α levels in cardiomyocytes after transfection of si *Xrcc6*. n = 4. **(E)** Representative images of intracellular ROS in cardiomyocytes transfected with si *Xrcc6*. Scale bar = 50 μm. **(F)** Quantitation of intracellular ROS fluorescence density. n = 4. **(G)** Protein levels of γH2A.X after transfection of si *Xrcc6*. n = 3. **(H)** Cell viability after overexpression of *Xrcc6*. n = 4. **(I)** The activity of LDH after overexpression of *Xrcc6*. n = 4. **(J, K)** Normalized IL-6 and TNF-α levels in cardiomyocytes after overexpression of *Xrcc6*. n = 4. **(L)** Representative images of intracellular ROS in cardiomyocytes transfected with *Xrcc6* overexpression plasmids. Scale bar = 50 μm. **(M)** Quantitation of intracellular ROS fluorescence density. n = 4. **(N)** Protein levels of γH2A.X after overexpression of *Xrcc6*. n = 3.

Using a primary cardiomyocyte culture model, we further investigated the role of *Xrcc6* in regulating H_22_ -induced cardiomyocyte injury. Knockdown of *Xrcc6* markedly reduced cardiomyocyte viability (**Figure 9A**) and significantly increased LDH release into the culture supernatant (**Figure 9B**). Moreover, *Xrcc6* knockdown substantially elevated intracellular levels of the pro-inflammatory cytokines IL-6 and TNF-α, as well as ROS production (**Figures 9C–F**), and further upregulated γH2A.X expression (**Figure 9G**). In contrast, *Xrcc6* overexpression mitigated H_22_ -induced cardiomyocyte injury, as evidenced by improved cell viability, reduced LDH release, decreased pro-inflammatory cytokine and ROS levels, and attenuated γH2A.X expression (**Figures 9H–N**).

#### 3.5.10 Knockdown of *Xrcc6* attenuates the cardioprotective effects of Fisetin

**Figure 10:**
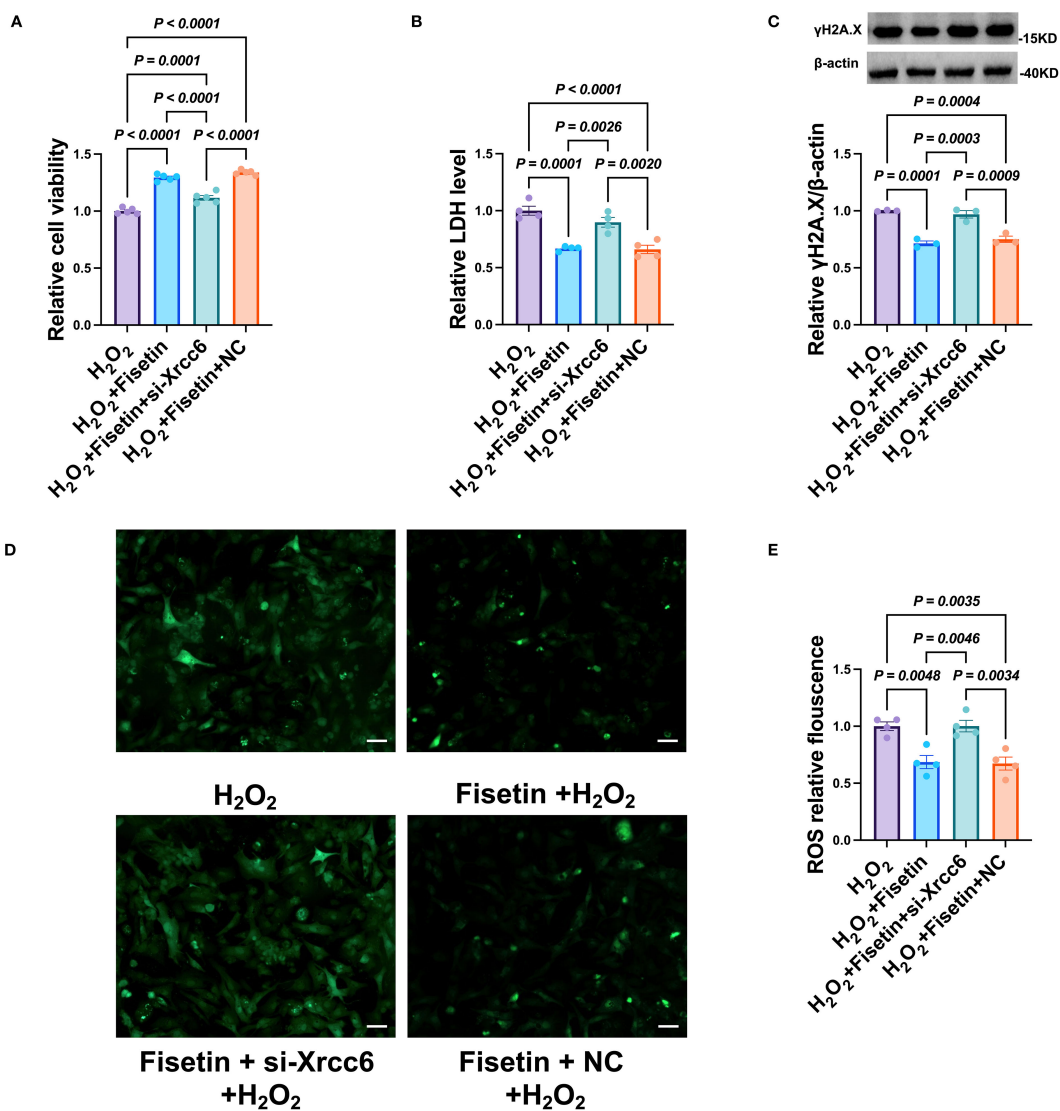
Knockdown of Xrcc6 abrogates the protective effects of Fisetin in H2O2-treated cardiomyocytes. **(A)** Cell viability after co-treatment of siRNA of *Xrcc6* and Fisetin. n = 4. **(B)** The activity of LDH after co-treatment of siRNA of *Xrcc6* and Fisetin. n = 4. **(C)** Protein levels of gamaH2A.X after co-treatment of siRNA of *Xrcc6* and Fisetin. n = 3. **(D)** Representative images of intracellular ROS in cardiomyocytes. Scale bar = 50 μm. **(E)** Quantitation of intracellular ROS fluorescence density. n = 4.

To further determine whether the cardioprotective effects of Fisetin are mediated by *Xrcc6*, we simultaneously knocked down *Xrcc6* while treating cardiomyocytes with Fisetin. *Xrcc6* knockdown significantly reduced the protective effect of Fisetin against H_22_ -induced cardiomyocyte injury (**Figure 10A**). Specifically, *Xrcc6* knockdown partially reversed the Fisetin-induced reductions in LDH release (**Figure 10B**) and γH2A.X expression (**Figure 10C**). In addition, *Xrcc6* knockdown abrogated the Fisetin-mediated suppression of intracellular ROS levels (**Figures 10D, E**). These findings indicate that *Xrcc6* plays a critical role in mediating the cardioprotective effects of Fisetin.

## 4 Discussion

Ischemia-reperfusion (I/R) injury, recognized as a “second hit” during the clinical management of acute coronary syndrome, remains inadequately understood at the molecular level, making it a focal point in cardiovascular research (53). Although revascularization can salvage jeopardized myocardial tissue, the ensuing oxidative stress and immune-inflammatory responses caused by reperfusion can exacerbate tissue damage (54). Therefore, elucidating the molecular mechanisms of I/R injury is crucial for the development of effective protective strategies. Previous studies have highlighted the central role of DNA damage, particularly double-strand breaks, in myocardial cell death and dysfunction during I/R injury (55). This study investigates the regulatory mechanism of the DNA repair factor *Xrcc6*, utilizing transcriptomics, single-cell sequencing, molecular simulations, and animal experiments to reveal its role in myocardial I/R injury and further explore the feasibility of Fisetin, a natural flavonoid, as a protective agent targeting *Xrcc6*.

Initially, we established a hydrogen peroxide (H_22_ )-induced oxidative stress model in myocardial cells. Transcriptomic analysis revealed a significant downregulation of multiple DNA repair genes, including *Xrcc6*, under simulated I/R conditions, suggesting severe DNA damage in myocardial cells during reperfusion with compromised repair capacity. As a key factor in the non-homologous end-joining (NHEJ) repair pathway, the expression changes in *Xrcc6* suggest the inhibition of NHEJ repair, promoting apoptosis or necrosis. Gene Ontology (GO) enrichment analysis further identified significant enrichment in pathways associated with DNA repair, oxidative stress response, and mitochondrial function—key pathological processes of I/R injury—providing a strong biological foundation for targeting *Xrcc6* in our study.

To explore the tissue-specific function of *Xrcc6*, we utilized publicly available single-cell transcriptomic data from the heart. Clustering analysis identified four major subtypes of myocardial cells, with the vCMs3 subtype predominating in the normal state but significantly decreasing after I/R. The gene expression profile of this subtype revealed high undifferentiated potential, enriched in biological processes related to myocardial development and cellular differentiation, suggesting its potential as a regenerative repair myocardial cell subtype. Notably, *Xrcc6* expression was significantly higher in the vCMs3 subtype compared to other subtypes, with its expression dynamically regulated along the differentiation trajectory. Trajectory analysis indicated that vCMs3 is positioned at the beginning of the myocardial cell differentiation pathway, which shifts under I/R injury, exhibiting transcriptional reprogramming driven by *Xrcc6*. This finding not only provides spatial and temporal insights into *Xrcc6*’s role in determining cell fate but also identifies a novel target for early intervention.

In addition to the cellular stress response in myocardial cells, I/R injury induces substantial changes in the composition and functional status of immune cells. CIBERSORT-based immune cell infiltration analysis revealed a significant increase in M0 macrophages and a decrease in M1 pro-inflammatory macrophages after I/R, suggesting a dynamic transition towards a reparative inflammatory response. Correlation analysis showed a positive correlation between *Xrcc6* expression and M0 macrophages, and a negative correlation with M1 macrophages, implying a regulatory role for *Xrcc6* in macrophage polarization. Further cell communication network analysis indicated a significant enhancement in signaling interactions between myocardial cells and macrophages under I/R conditions, with the Laminin pathway being highly activated as a key signaling axis. These findings provide a new hypothesis framework: *Xrcc6* not only serves as a core factor in DNA repair within myocardial cells but may also participate in intercellular communication and remodeling of the immune microenvironment, acting as a crucial molecular hub linking cell damage and tissue repair.

Building upon our deep exploration of *Xrcc6*’s function, we further investigated its potential as a drug target. Fisetin, a widely distributed and pharmacologically well-characterized natural flavonoid, has been shown to possess antioxidant, anti-apoptotic, and anti-inflammatory properties in various disease models (56–58). Molecular docking and dynamics simulations confirmed that Fisetin binds stably to the active site of *Xrcc6*, with a binding free energy of -7.55 kcal/mol. The binding process involves multiple hydrogen bonds and hydrophobic interactions, maintaining the stability of the complex. Key residues of *Xrcc6*, such as PHE-200 and LYS-331, exhibited strong binding adaptability and energy contributions, further supporting *Xrcc6* as a potential target for Fisetin. The RMSD, Rg, and SASA analyses indicated that the complex remained stable throughout the simulation, with no structural dissociation or energy disruption, providing a theoretical basis for *in vivo* functional intervention.

Animal experiments further validated the mechanism derived from molecular simulations. Fisetin intervention significantly improved myocardial function in I/R mice, including enhanced ejection fraction (EF) and fractional shortening (FS), and reduced left ventricular end-diastolic diameter (LVIDd). Biochemical markers showed that Fisetin effectively lowered LDH and CK-MB levels, indicating significant protection against myocardial cell injury. ELISA analysis of inflammatory cytokines revealed that Fisetin markedly inhibited the release of IL-6 and TNF-α, demonstrating its potent anti-inflammatory effects. More importantly, Fisetin significantly upregulated *Xrcc6* expression in myocardial tissue and reduced the accumulation of DNA damage marker γH2A.X, confirming that its protective mechanism is closely related to the enhancement of DNA repair function. Taken together, these *in vitro* and *in vivo* studies establish a comprehensive intervention chain: “Fisetin—*Xrcc6*—DNA repair—Immune regulation.

Although this study has made significant progress in elucidating the mechanisms and pharmacological validation, several limitations remain. First, our research primarily relies on expression associations and simulation data, and has yet to directly validate the pathogenic and protective roles of *Xrcc6* in I/R through gene knockout or overexpression in cell models or animal models. Future studies could establish myocardium-specific *Xrcc6* knockout mouse models to clarify its functional boundaries in myocardial cells and the immune system. Second, while Fisetin is a natural small molecule, its pharmacokinetic properties and myocardial targeting *in vivo* remain to be further investigated and optimized. Given its strong binding affinity, combining Fisetin with drug delivery systems, such as nanomicelles or liposomal carriers, may further enhance its bioavailability and tissue selectivity.

*Xrcc6* (Ku70) is a core component of the DNA non-homologous end joining (NHEJ) pathway. Together with XRCC5 (Ku80), it forms a heterodimer that recognizes DNA double-strand breaks (DSBs) and recruits DNA-PKcs and other repair enzymes to maintain genomic stability. Loss of *Xrcc6* function impairs DSB repair, leading to genomic instability and an increased risk of malignant transformation.

Emerging evidence suggests that *Xrcc6* is also involved in immune-metabolic regulation via TLR4 signaling. TLR4 activation upregulates *Xrcc6* expression, whereas TLR4 deficiency downregulates *Xrcc6*, resulting in the accumulation of DNA damage, increased ROS levels, and suppression of autophagy/senescence programs, ultimately promoting hepatocarcinogenesis (59). This pathway highlights the possibility that *Xrcc6* may indirectly influence macrophage polarization by modulating DNA repair capacity and oxidative stress status. Excessive ROS accumulation is a well-established driver of macrophage polarization toward the pro-inflammatory M1 phenotype. Consistent with this, we observed that *Xrcc6* silencing impaired ROS clearance and induced the secretion of pro-inflammatory cytokines such as IL-1β and TNF-α. Conversely, *Xrcc6* overexpression markedly reduced ROS levels and suppressed inflammatory responses.

Collectively, these findings suggest that *Xrcc6* counteracts M1 polarization by promoting efficient DSB repair, thereby reducing ROS production and downstream pro-inflammatory signaling cascades. Thus, *Xrcc6* integrates the “DNA repair–oxidative stress” axis to regulate macrophage polarization balance, positioning it as a critical node that links genomic stability to the regulation of the immune microenvironment.

In conclusion, this study, through multi-omics and multi-level analysis, unveils the multifaceted roles of *Xrcc6* in myocardial I/R injury, confirming that *Xrcc6* is involved not only in DNA damage repair but also in the regulation of myocardial cell fate and immune microenvironment remodeling. As a natural regulator of *Xrcc6*, Fisetin demonstrates clear targeting and protective effects, making it a promising candidate for the intervention of myocardial I/R injury.

## 5 Conclusion

This study systematically elucidates the pivotal role of the DNA repair factor *Xrcc6* in ischemia-reperfusion (I/R) injury, demonstrating that it is involved not only in the repair of DNA double-strand breaks in myocardial cells but also in regulating the fate transitions of myocardial cell subtypes and remodeling the immune microenvironment. Using transcriptomic and single-cell transcriptomic technologies, we observed a significant downregulation of *Xrcc6* in response to I/R. Notably, *Xrcc6* was highly expressed in the regenerative myocardial subtype vCMs3, and its expression was closely linked to its differentiation trajectory. Immune analysis further revealed a strong correlation between *Xrcc6* expression and macrophage polarization status, suggesting that *Xrcc6* may coordinate the damage-repair process through intercellular signaling pathways.

We also confirmed the molecular basis for Fisetin, a natural flavonoid, as a potential targeted modulator of *Xrcc6*. Molecular docking and dynamics simulations demonstrated that Fisetin can stably bind to the active site of *Xrcc6*. Animal experiments verified that Fisetin significantly improved cardiac function, reduced myocardial injury and inflammation, and enhanced the *Xrcc6*-mediated DNA repair pathway.

In summary, *Xrcc6* is a critical regulatory factor in myocardial I/R injury, and Fisetin offers protection of myocardial cell structure and function through targeting *Xrcc6*. This study not only expands the understanding of the molecular mechanisms underlying I/R injury but also provides a theoretical foundation and practical basis for developing natural drug intervention strategies targeting DNA repair pathways, with significant clinical translation potential.

## Data Availability

The RNA sequencing data generated in this study have been deposited in public repositories. Raw RNA-seq data are available in the NCBI Sequence Read Archive (SRA) under accession number PRJNA1282757. Processed data, including raw counts and FPKM matrices, are deposited in the NCBI Gene Expression Omnibus (GEO) under accession number GSE301051.

## Supplementary material

Supplementary Figure 1 Single-cell transcriptomic analysis reveals distinct cardiac cell types and gene co-expression module characteristics. **(A)** UMAP projection showing the clustering of cardiac cell populations. A total of 10 distinct cell types were identified. **(B)** Bar plot comparing the relative proportions of each cell type between Sham and I/R groups. **(C)** Dot plot illustrating the expression of canonical marker genes across identified cell types. **(D)** Hierarchical clustering dendrogram of gene modules constructed using high-dimensional weighted gene co-expression network analysis (hdWGCNA). Modules are color-coded. **(E)**: Correlation heatmap among gene co-expression modules (M1–M5). Dark purple ellipses represent strong positive correlations, while green indicates negative correlations. Color intensity reflects the magnitude of correlation coefficients. **(F)** Bubble plot showing the association between gene modules and ventricular cardiomyocyte subtypes. Bubble size denotes the proportion of cells expressing module genes; color indicates average expression level. **(G)** Network diagram illustrating functional interactions among genes within a selected module.

Supplementary Figure 2 Batch effect correction of snRNA-seq data using Harmony. **(A)** UMAP projection of all cells from Sham and I/R samples before batch correction, showing separation partially driven by technical batch effects. **(B)** UMAP projection after Harmony integration, demonstrating that batch-associated variation was effectively minimized while preserving biologically meaningful clustering of diverse cardiac cell populations.

Supplementary Figure 3 Comparative Analysis of Cardiomyocyte Developmental Trajectories and Condition-Specific Signaling Pathways Using Multiple Inference Algorithms. **(A)** Developmental trajectory reconstruction using the Slingshot algorithm, showing distinct lineage branches, pseudotime progression, and data projection along inferred differentiation paths. **(B)** Left: Partition-based graph abstraction (PAGA) latent transition neighborhood network (LTNN) depicting putative developmental relationships among cardiomyocyte subtypes (vCMs1–vCMs4); Middle: pseudotime distribution along inferred trajectories; Right: entropy distribution across cells, indicating levels of transcriptional uncertainty. **(C)** Left: Pseudotime trajectory network integrated from the Atlas dataset, colored by pseudotime position; Middle: clustering structure of cardiomyocyte subtypes; Right: projected topology of differentiation trajectories, inferred under parameters *K* = 15 and 50 principal components. **(D)** Comparative analysis of condition-conserved and condition-specific signaling pathways. Left: differential enrichment scores of signaling pathways between Sham and I/R groups; Right: bar plots showing activity scores of selected pathways, highlighting differences in condition-specific signaling activation.
