# Supplementary figures and images for: Xrcc6 coordinates cardiomyocyte repair and immune regulation in myocardial ischemia-reperfusion injury: Fisetin as a therapeutic modulator
Source: Front Immunol. 2025 Sep 17;16:1653738. doi: 10.3389/fimmu.2025.1653738 (PMC12483892; doi:10.3389/fimmu.2025.1653738)

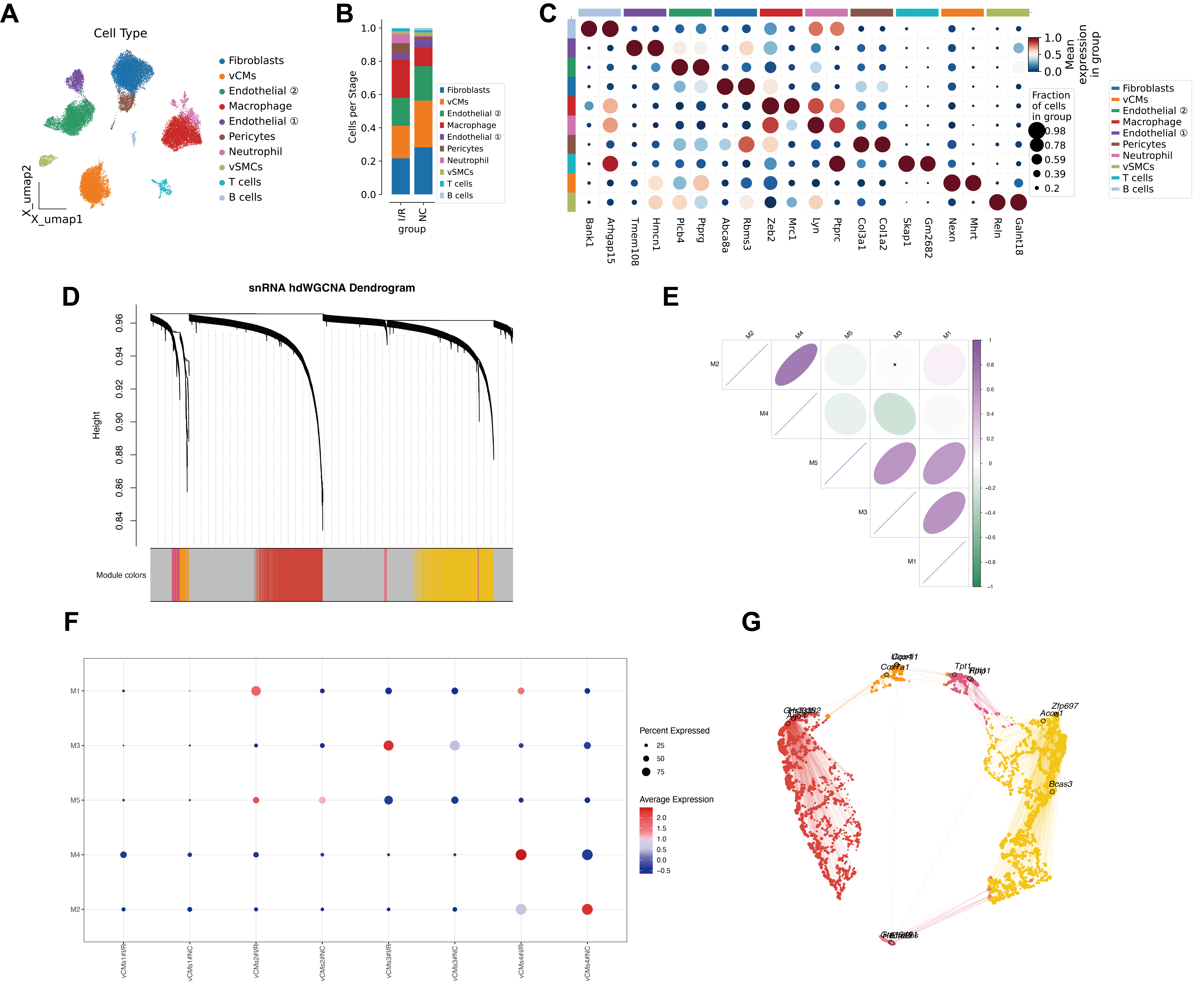

Supplement: Supplementary Figure 1 — Single-cell transcriptomic analysis reveals distinct cardiac cell types and gene co-expression module characteristics. (A) UMAP projection showing the clustering of cardiac cell populations. A total of 10 distinct cell types were identified. (B) Bar plot comparing the relative proportions of each cell type between Sham and I/R groups. (C) Dot plot illustrating the expression of canonical marker genes across identified cell types. (D) Hierarchical clustering dendrogram of gene modules constructed using high-dimensional weighted gene co-expression network analysis (hdWGCNA). Modules are color-coded. (E): Correlation heatmap among gene co-expression modules (M1–M5). Dark purple ellipses represent strong positive correlations, while green indicates negative correlations. Color intensity reflects the magnitude of correlation coefficients. (F) Bubble plot showing the association between gene modules and ventricular cardiomyocyte subtypes. Bubble size denotes the proportion of cells expressing module genes; color indicates average expression level. (G) Network diagram illustrating functional interactions among genes within a selected module. [file Image1.jpeg]

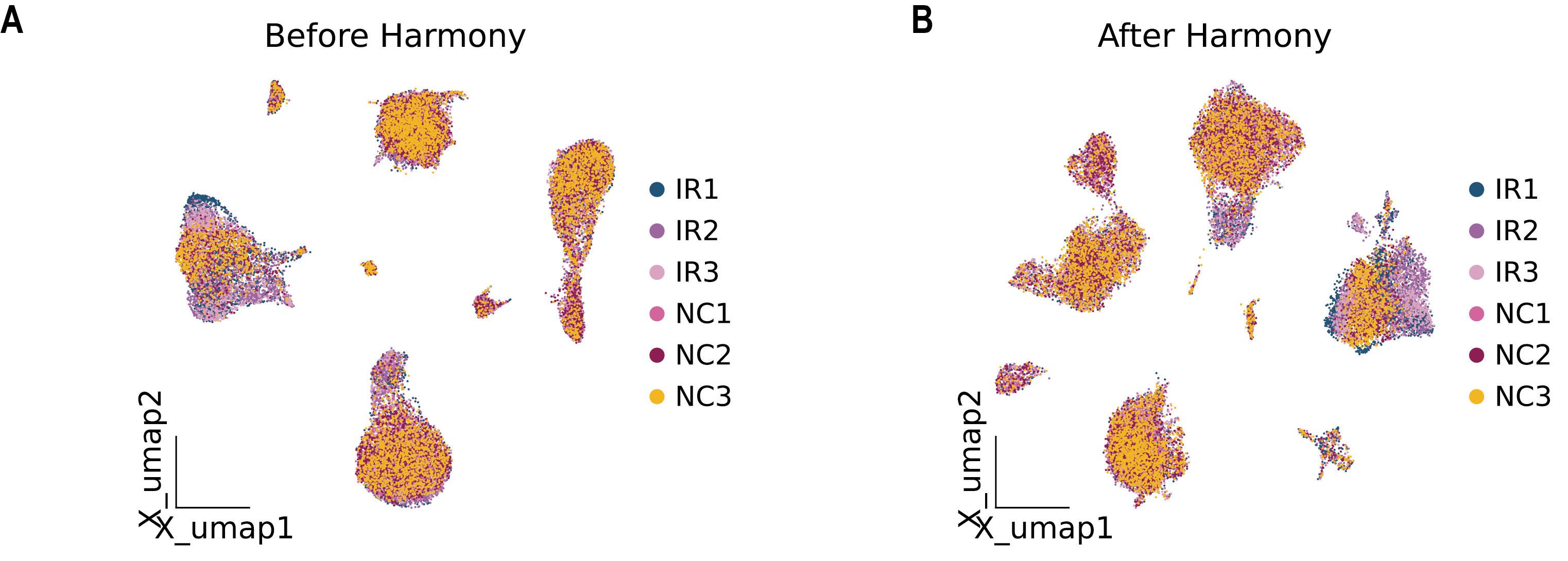

Supplement: Supplementary Figure 2 — Batch effect correction of snRNA-seq data using Harmony. (A) UMAP projection of all cells from Sham and I/R samples before batch correction, showing separation partially driven by technical batch effects. (B) UMAP projection after Harmony integration, demonstrating that batch-associated variation was effectively minimized while preserving biologically meaningful clustering of diverse cardiac cell populations. [file Image2.jpeg]

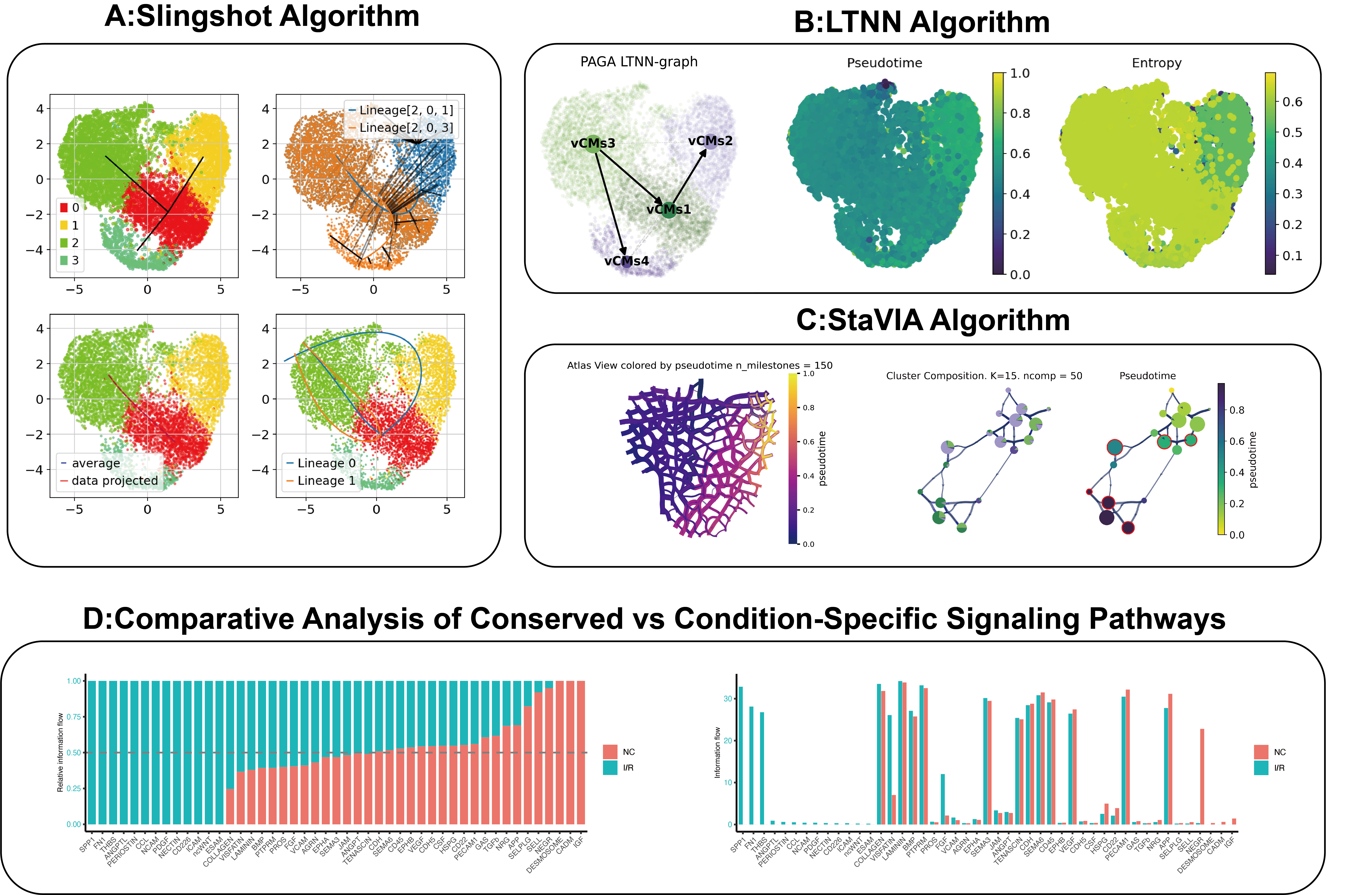

Supplement: Supplementary Figure 3 — Comparative Analysis of Cardiomyocyte Developmental Trajectories and Condition-Specific Signaling Pathways Using Multiple Inference Algorithms. (A) Developmental trajectory reconstruction using the Slingshot algorithm, showing distinct lineage branches, pseudotime progression, and data projection along inferred differentiation paths. (B) Left: Partition-based graph abstraction (PAGA) latent transition neighborhood network (LTNN) depicting putative developmental relationships among cardiomyocyte subtypes (vCMs1–vCMs4); Middle: pseudotime distribution along inferred trajectories; Right: entropy distribution across cells, indicating levels of transcriptional uncertainty. (C) Left: Pseudotime trajectory network integrated from the Atlas dataset, colored by pseudotime position; Middle: clustering structure of cardiomyocyte subtypes; Right: projected topology of differentiation trajectories, inferred under parameters K = 15 and 50 principal components. (D) Comparative analysis of condition-conserved and condition-specific signaling pathways. Left: differential enrichment scores of signaling pathways between Sham and I/R groups; Right: bar plots showing activity scores of selected pathways, highlighting differences in condition-specific signaling activation. [file Image3.jpeg]
